# Rapid development and field evaluation of a portable CRISPR-based assay for Mpox during the 2025 Sierra Leone outbreak

**DOI:** 10.1038/s41467-026-74034-8

**Published:** 2026-06-06

**Authors:** Nisha Gopal, Tsion Abay, Carolyn Payne, Michael Gomez, Maariam Manjia Rogers, Ibrahim Umaru Fofanah, Tiangay P. M. S. Kallon, Mohamed S. Kamara, Ho-Jun Suk, John Demby Sandi, Taylor Brock-Fisher, Elyse Stachler, Lao-Tzu Allan-Blitz, David J. Roach, Marietou F. Paye, Colby Wilkason, Donald S. Grant, Al Ozonoff, Pardis C. Sabeti

**Affiliations:** 1 Broad Institute of Massachusetts Institute of Technology and Harvard, Boston, Massachusetts USA; 2 Harvard Graduate Program in Biological and Biomedical Science, Boston, MA USA; 3 William & Mary, Williamsburg, VA USA; 4 Kenema Government Hospital, Ministry of Health, Kenema, Sierra Leone; 5 DxLab Inc., Somerville, Massachusetts, USA; 6 College of Medical Sciences, Njala University, Freetown, Sierra Leone; 7 Department of Organismic and Evolutionary Biology, Harvard University, Cambridge, Massachusetts USA; 8 Division of Global Health Equity, Brigham and Women’s Hospital, Boston, MA USA; 9 Division of Infectious Diseases, Brigham and Women’s Hospital, Boston, MA USA; 10 College of Medicine and Allied Health Sciences, University of Sierra Leone, Freetown, Sierra Leone; 11 Department of Clinical Sciences, Liverpool School of Tropical Medicine, Liverpool, UK; 12 Harvard Medical School, Boston, Massachusetts USA; 13 Boston Children’s Hospital, Boston, Massachusetts USA; 14 Department of Immunology and Infectious Disease, Harvard T.H. Chan School of Public Health, Boston, Massachusetts USA; 15 Howard Hughes Medical Institute, Chevy Chase, Maryland USA

**Keywords:** CRISPR-Cas systems, Viral infection, Biosensors, Infectious-disease diagnostics

## Abstract

The large 2025 Mpox clade IIb outbreak in Sierra Leone underscores the urgent need for portable, low-cost diagnostics in decentralized settings. While CRISPR-based assays offer high sensitivity and flexibility, their deployment during active outbreaks remains limited. Here we show the rapid development and field evaluation of Mpox SHINE, a CRISPR–Cas13 assay that integrates lyophilized reagents, ambient-temperature lysis, and automated fluorescence detection on the portable DxHub device. The assay achieves analytical sensitivity down to 10 copies/µL. Clinical validation in Sierra Leone, using 56 clinical specimens, confirms complete concordance with qPCR, demonstrating 100% sensitivity and 100% specificity. Crucially, Mpox SHINE also detects the virus directly from unextracted lesion swabs while maintaining 100% sensitivity and specificity. The mean time-to-result is fast, averaging 11.4 minutes for extracted samples and 27.9 minutes for unextracted samples. These findings demonstrate that CRISPR-based diagnostics translate quickly from genomic sequence to clinically validated, deployable tools within a single outbreak window.

## Abstract (web summary)

The Mpox outbreak in Sierra Leone necessitated fast, decentralized testing. Here, authors developed and validated Mpox SHINE, a portable CRISPR diagnostic that delivered 100% accurate results on patient samples in under 30 minutes, showing these tools can be deployed rapidly during an outbreak.

## Introduction

In early 2025, Sierra Leone faced a fast-moving outbreak of mpox clade IIb that infected thousands and strained the country’s public health system. By July, more than 4000 confirmed cases had been reported, demonstrating both the scale of the crisis and the risk of wider regional spread^1–5^. Mpox is caused by the mpox virus (MPXV), an enveloped double-stranded DNA Orthopoxvirus in the Poxviridae family, and spreads through both animal-to-human and human-to-human transmission, often via close contact with lesions and other bodily fluids^6^. This crisis followed on the heels of successive mpox resurgences, including a global multi-country outbreak of clade IIb in 2022, and a surge of clade I, with lineage Ib, across central Africa in 2024, when the World Health Organization (WHO) declared mpox a public health emergency of international concern^7,8^.

Despite the escalating global health threat of mpox, diagnostics remain constrained worldwide^9^. Testing depends almost entirely on qPCR, or expensive cartridge-based systems^10^, performed in a handful of centralized laboratories. Although qPCR is highly sensitive, it requires costly infrastructure, specialized equipment, and trained personnel, limiting accessibility in rural districts experiencing the heaviest caseloads. As a result, frontline clinics are left without access to timely results, a problem exacerbated by severe testing shortages that create backlogs and multi-day delays at the very moment when detection is most critical. Unlike other viral diseases, rapid antibody-based assays (rapid diagnostic tests, RDTs) for mpox are not widely available, nor are any clinically approved portable tests for mpox available for distribution at decentralized sites throughout Sierra Leone. The result is a widening diagnostic gap: community surveillance stalls, sample turnaround is delayed, and the outbreak spreads faster than the system can respond.

More broadly, a critical gap persists between the rapid generation of pathogen genomic data and the ability to translate that information into deployable, field-validated diagnostics during an active outbreak. While molecular assays can often be designed within days, their integration into robust, scalable, and clinically usable systems remains a major bottleneck for real-time outbreak response.

SHINE (Streamlined Highlighting of Infections to Navigate Epidemics^11^ is an established CRISPR-based nucleic acid detection strategy that provides a promising solution for diagnostic deployment gaps^12^. By combining recombinase polymerase amplification (RPA)-based isothermal nucleic acid amplification with programmable Cas13a-mediated detection, SHINE can achieve sensitivity and specificity comparable to qPCR^13,14^. The use of established artificial intelligence (AI)-guided assay design tools like ADAPT (Activity-informed Design with All-inclusive Patrolling of Targets) enables the design of Cas13a-specific guide RNAs for viral genome detection, optimized for high predicted activity levels and maximal genome conservation^15^. In addition, lyophilized reagents, ambient-temperature lysis, and simplified workflows reduce the need for equipment or cold chain, allowing assays to be rapidly distributed and performed at the point of need. Together, these features support rapid translation of CRISPR diagnostics from design to field use on outbreak-relevant timescales. Together, these features create a foundation for rapid translation from sequence-informed assay design to decentralized testing. While other CRISPR-based point-of-care platforms have been developed in recent years (see Supplementary Table 2), our work demonstrates end-to-end deployment under real-world outbreak conditions.

Here we demonstrate the rapid development, integration, and field evaluation of Mpox SHINE using the portable DxHub platform (DxLab Inc., Somerville, MA, USA) during the Sierra Leone clade IIb outbreak. In response to the active outbreak, we advanced from assay design to on-site validation in Sierra Leone through a coordinated development process, encompassing primer and crRNA design, ambient lysis optimization, lyophilization, portable device integration, and clinical sample evaluation at Kenema Government Hospital. This work demonstrates that a CRISPR-based diagnostic system can be rapidly mobilized from genomic sequence to in-country clinical sample validation during an active outbreak. The sections that follow describe assay design, analytical performance on synthetic targets, device integration, and evaluation on both extracted and minimally processed clinical samples, achieved within a single outbreak window.

## Results

### Design, optimization, and validation of a SHINE assay for Mpox Clade IIb

To rapidly respond to the 2025 Sierra Leone mpox outbreak, we first developed an Mpox SHINE assay and performed analytical validation using synthetic targets.

**Fig. 1:**
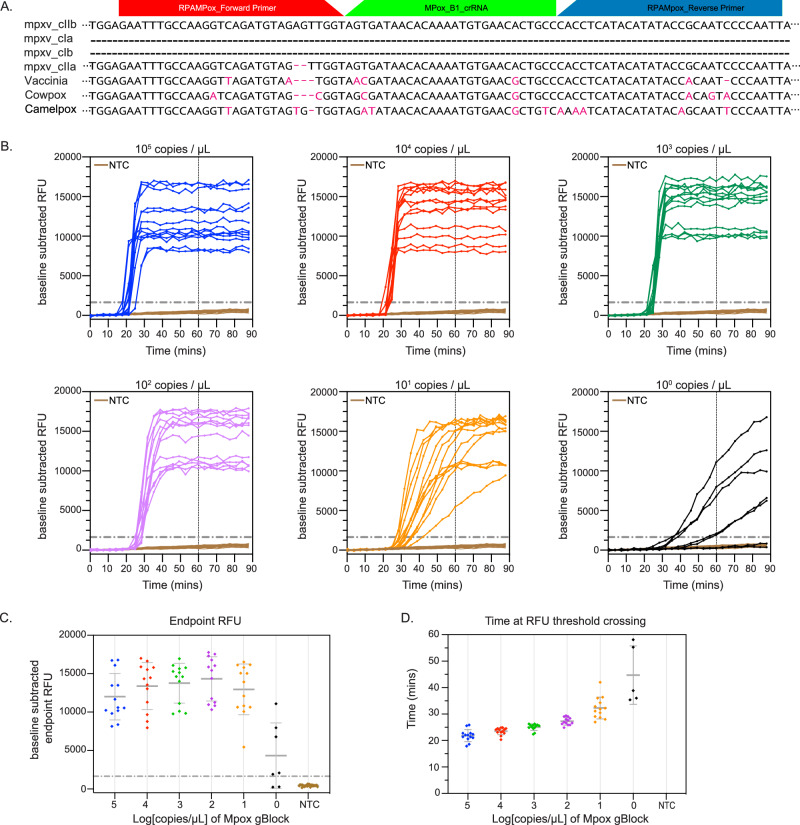
Mpox SHINE assay design and analytical performance on a plate reader. **A** Schematic of the Mpox target locus. Forward and reverse RPA primers (red/blue) and the crRNA spacer (green) are shown aligned to the MPXV clade IIb genome (“Pathoplexus | Mpox Virus Genomes,” n.d.) together with MPXV clade IIa (97.5% identity; “Pathoplexus | Mpox Virus Genomes,” n.d.) and representative Orthopoxvirus genomes: vaccinia (NC_006998.1, 91.3% identity), cowpox (NC_003663.2, 91.3% identity), and camelpox (AY009089.1, 86.3% identity). Bases that are mismatched from MPXV clade IIb are in pink. The amplicon lies in a conserved region of clade II Mpox, with primer and guide sequences listed in Supplementary Table 1. **B** Plate reader measured fluorescence kinetics (baseline-subtracted RFU; baseline = mean RFU from 1–4 min) for a 10-fold dilution series of synthetic target (10^5^ to 10^0^ copies /µL of reaction) and NTCs. Solid lines indicate individual replicates of Mpox SHINE reactions, and the dotted horizontal line indicates the fixed RFU threshold (NTC mean at 60 min + 10 SD) used for result calling. **C** Endpoint RFU at 60 min for the same datasets as (**B**), plotted as individual technical replicates (pooled from two experiments) for 10^1^–10^5^ copies/µL (*n* = 13), 10^0^ copies/µL (*n* = 7), and NTCs (*n* = 32). Horizontal bars denote the mean, and error bars denote ±1 SD across technical replicates. The dotted horizontal line indicates the fixed RFU threshold (NTC mean at 60 min + 10 SD). Reactions exceeding this threshold were classified as positive, as described in the “Methods”. **D** Time to RFU threshold crossing for the same datasets, with all replicates corresponding to the same datasets in panel (**B**, **C**). Horizontal bars denote the mean, and error bars denote ±1 SD across technical replicates. Source data are provided as a Source Data file.

Using ADAPT (Activity-informed Design with All-inclusive Patrolling of Targets), an AI-driven assay design framework, we identified a highly conserved amplicon as a suitable target for mpox clade IIb detection. From 33 newly sequenced genomes generated at Kenema Government Hospital in April–May 2025^16^ (Supplementary Data 2), we designed forward and reverse RPA primers and a Cas13 crRNA with complete coverage (zero mismatches) across these sequences (Supplementary Table 1, Supplementary Data 2). The ADAPT-derived amplicon was an 88 bp segment in the 3′ variable region between gp161 and gp162, upstream of the inverted terminal repeat (NC_063383) (Fig. 1A, Supplementary Table 1). This locus is fully conserved across clade IIb and highly conserved in clade IIa (97.6%) but absent from clade I (Ia/Ib) due to deletions observed in our alignment. In comparison, sequence identity to other orthopoxviruses was lower (vaccinia 91.3%, cowpox 91.3%, camelpox 86.3%), reflecting divergence from non-mpox orthopoxes while retaining broad coverage of outbreak-relevant lineages.

We optimized the Mpox SHINE assay to maximize performance and sensitivity using a synthetic double-stranded DNA target containing the selected amplicon (Supplementary Table 1). We tuned primer concentration, primer composition, and magnesium concentration systematically to improve signal (Supplementary Fig. 1). We performed all reactions using lyophilized SHINE reagents containing the newly designed Mpox primers and crRNA (Fig. 1A). To assess analytical sensitivity, we tested a tenfold dilution series of a synthetic dsDNA mpox target sequence from 10^5^ to 10^0^ copies/µL at 38 °C. We monitored fluorescence on a Biotek Cytation 5 plate reader every 20 s for at least 60 min (Fig. 1B, C).

The optimized assay achieved highly sensitive detection, with a limit of detection (LOD) between ~1 and ~10 copies/µL, with complete detection at 10 copies/µL. Using a fixed fluorescence threshold (1644.9 RFU) defined as the mean of no template controls (NTC) plus 10 standard deviations (SD), we detected ~1 copy/µL in 5 of 7 replicates (71.4%, 95 % CI 35.9–91.8) and ~10 copies/µL in 13 of 13 replicates (100 %, 95 % CI 77.2–100.0). These results demonstrate consistent single-digit copy sensitivity using laboratory equipment.

We next asked whether the assay could also deliver a rapid time to detection suitable for field use. Time to detection was defined as the point at which fluorescence signal crossed the fixed threshold, and it was inversely related to input copy number (Fig. 1D). At high input levels (10^5^–10^2^ copies/µL), all replicates crossed the threshold within approximately 21–28 min, while the lowest-input samples near the detection limit (10^1^–10^0^ copies/µL) were detected within 27–45 min. No amplification was observed in NTCs over the 60-min assay window. Together, these results demonstrate the assay’s rapid and reliable detection across a clinically relevant range of viral inputs.

### Integration of Mpox SHINE on the DxHub portable isothermal device

**Fig. 2:**
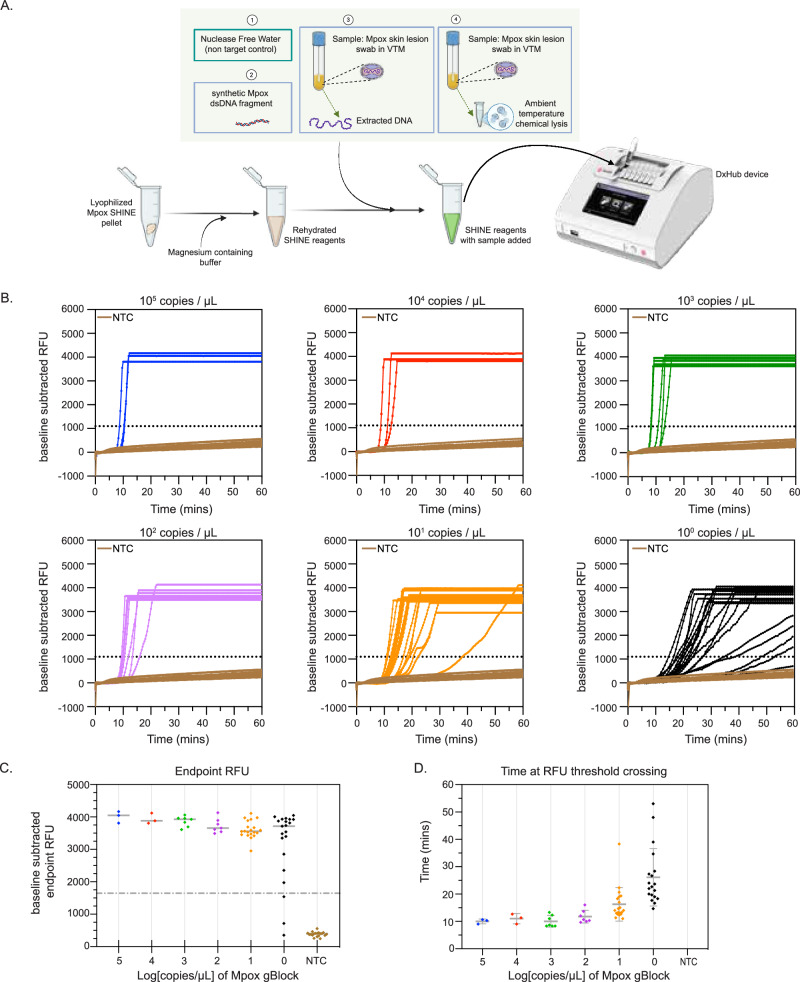
Device integration, workflow, and on-device analytical performance. **A** Workflow for running Mpox SHINE on the DxHub. A lyophilized single-reaction pellet is rehydrated with magnesium-containing buffer, the sample is added (synthetic dsDNA target, water as a no-template control, extracted clinical DNA, or chemically lysed mpox specimen), and the tube is loaded into the device for incubation at 38 °C along with concurrent fluorescent signal acquisition. VTM = viral transport media. Created in BioRender. Gopal, N. (2026) https://BioRender.com/64nm3th. **B** On-device fluorescence kinetics (baseline-subtracted RFU; baseline = mean RFU from 1–4 min) for a 10-fold dilution series of target (10^5^ to 10^0^ copies /µL of reaction) and NTCs. Solid lines indicate individual technical replicates of Mpox SHINE reactions at different target concentrations, and the dotted horizontal line indicates the fixed RFU threshold (NTC mean at 60 min + 10 SD) used for result calling. **C** Endpoint RFU at 60 min for the same datasets as (**B**), plotted as individual technical replicates (pooled from 4 experiments). Replicate counts: 10^0^ copies /µL (*n* = 21), 10^1^ copies /µL (*n* = 20), 10^2–3^ copies /µL (*n* = 7), 10^4–5^ copies /µL (*n* = 3), and NTCs (*n* = 19). Horizontal bars denote the mean, and error bars denote ±1 SD across technical replicates. The dotted horizontal line indicates the fixed RFU threshold (NTC mean + 10 SD) used for reaction classification as described in the “Methods”. **D** Time to RFU threshold crossing for the same datasets, with all replicates corresponding to the same datasets in panel (**B**, **C**). Horizontal bars denote the mean, and error bars denote the standard deviation. Source data are provided as a Source Data file.

With the Mpox SHINE assay benchmarked on a plate reader, we next integrated it onto a portable isothermal device to evaluate performance in a decentralized testing context. The DxHub (DxLab Inc., Somerville, MA) is a compact device that provides isothermal incubation and dual channel real time fluorescence detection (Fig. 2A). Achieving a quantifiable fluorescence readout in real time is a key feature of SHINE, however, most prior SHINE studies relied on lateral flow strips, which provide a simple visual readout but are non-quantitative and less amenable to monitoring reaction kinetics^11,17–19^. By contrast, the DxHub, designed for decentralized surveillance use, enables quantitative fluorescence measurement, real-time kinetic monitoring, and automated thresholding with a simple three-step workflow requiring fewer than five minutes of hands-on time, enabling more objective data interpretation and result calling even in decentralized, low-infrastructure settings (Fig. 2A).

We benchmarked Mpox SHINE performance on the DxHub using a serial dilution of the synthetic mpox DNA (Fig. 2b) to evaluate whether assay performance observed on a standard laboratory plate reader would be retained following translation to this portable platform. Using a fixed fluorescence threshold defined as the mean of NTCs plus 10 SD (1097.5 RFU), we detected ~1 copy/µL in 19 of 21 replicates (90.5%; 95% CI 71.1–97.3%) and ~10 copies/µL in 20 of 20 replicates (100.0%; 95% CI 83.9–100.0%). Reaction positivity was determined relative to this platform-specific threshold rather than absolute fluorescence intensity. This yielded an analytical LOD between ~1 and ~10 copies/µL for this assay configuration, with complete detection at 10 copies/µL, consistent with results obtained using the plate reader configuration (Fig. 2B, C).

We next evaluated assay time to detection on the DxHub (Fig. 2D). Reactions just above the LOD were detected in an average of 16.3 ± 6.1 min, with higher-input samples detected in under 15 min. Compared to the plate reader measurements (Fig. 1D), the DxHub-based reactions resulted in earlier threshold crossings, likely reflecting platform-specific differences in fluorescence acquisition. Because fluorescence measurements are instrument-dependent, RFU values and threshold-crossing times are not directly comparable across platforms. Together, these results demonstrate that Mpox SHINE performs robustly after translation to a portable isothermal platform, while maintaining high analytical sensitivity and rapid time-to-detection (Figs. 1C, 2C).

### Mpox SHINE performance on extracted and minimally processed clinical samples

**Fig. 3:**
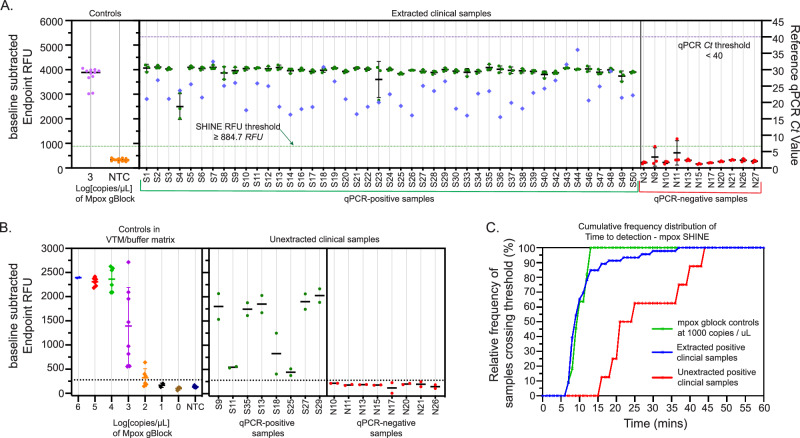
Clinical performance of Mpox SHINE on extracted and unextracted clinical samples. **A** An endpoint fluorescence of Mpox SHINE at 60 min (baseline-subtracted RFU; left y-axis) for controls and extracted clinical samples. The left panel shows controls (Mpox target in lavender [*n* = 11] and NTCs in orange [*n* = 14]). Middle panel displays qPCR-positive clinical samples (green, *n* = 45) and right panel shows qPCR-negative clinical samples are shown in the right panel (red, *n* = 11). For each sample, the mean ± SD across 3 technical replicates is shown, along with individual values. Blue diamonds represent corresponding confirmatory qPCR *Ct* values (right y-axis). The dotted green line indicates RFU threshold (mean of NTCs + 10SD). **B** Endpoint fluorescence of Mpox SHINE at 60 min (baseline-subtracted RFU; left y-axis) for controls and unextracted clinical samples. Control samples in matrix (VTM + saliva lysis buffer) are shown on the left with a 10-fold dilution series of the target from 10^6^ to 10^0^ copies /µL of reaction. Replicate counts: 10^0–1^ copies /µL (*n* = 2), 10^2^ copies /µL (*n* = 7), 10^3^ copies /µL (*n* = 8), 10^4–5^ copies /µL (*n* = 6), 10^6^ copies /µL (*n* = 2), and NTCs (*n* = 8). qPCR-positive unextracted clinical samples are shown in the middle (green symbols, *n* = 8 positive samples); qPCR-negative unextracted clinical samples are shown in the right panel (red symbols, *n* = 8 negative samples). For each Mpox clinical sample, the mean across 2 technical replicates is shown, along with the corresponding individual replicate values. The dotted black horizontal line marks the fixed RFU threshold based on the mean of NTCs shown on the left side of Panel B, at 60 min + 10 SD. **C** Cumulative frequency distribution of time-to-result for Mpox SHINE on the DxHub. Time-to-detection was defined as the first time point at which baseline-subtracted RFU crossed the predefined threshold, and for each sample, the mean across replicates was used. Curves show extracted qPCR-positive clinical samples (blue, *n* = 45), unextracted qPCR-positive clinical samples (green, *n* = 8), and 1e3 copies/µL target positive controls (red, *n* = 11). Source data are provided as a Source Data file.

Having established the analytical performance of Mpox SHINE in controlled settings, we next evaluated its performance on patient samples in the field at Kenema Government Hospital in Sierra Leone. We analyzed 56 lesion swabs collected in viral transport medium from patients and close contacts. Confirmatory qPCR on freshly extracted DNA identified 45 positives (Ct 15–36) and 11 negatives (Fig. 3A, Supplementary Data 1), which we used as the reference standard. We assessed sensitivity, specificity, and time to detection on the DxHub via two complementary experiments: one using extracted DNA from all specimens tested in triplicate to assess reproducibility, and one using chemically lysed, unextracted swabs tested in duplicate to probe performance in a simplified workflow.

On extracted samples, Mpox SHINE results closely tracked the qPCR reference standard, yielding a sensitivity of 100% (95% CI 92.1–100.0%) and a specificity of 100% (95% CI 74.1–100.0%) (Fig. 3A). All 45 qPCR-positive samples were positive in 3/3 replicates. Of the 11 qPCR-negative samples, 9 were negative in 3/3 replicates. The remaining 2 were negative in 2 of 3 replicates; in both cases, a single replicate crossed the fluorescence threshold late in the run, producing low-level amplification that did not replicate across repeat reactions (Supplementary Fig. 2). Because SHINE signal kinetics are proportional to input viral load, such weak late signals may reflect borderline or slow amplification. Thus, we applied a predefined majority rule criterion (2 of 3 replicates), which preserved specificity without masking potential low-level positives. In practice, such cases could be resolved through reflex testing, threshold adjustment, or confirmatory retesting workflows. Together, these results highlight the robustness and interpretability of Mpox SHINE across replicate runs and sample types.

Mpox SHINE delivered fast results across extracted clinical samples (Fig. 3c). The assay detected positives in an average of 11.4 ± 6.2 min, with 85% detected within 15 min and 100% within 37 min, consistent with the positive control kinetics (Supplementary Fig. 5A). Time to detection tended to increase with higher qPCR *Ct* values, but the Pearson correlation was weak and not statistically significant, and the Spearman correlation showed a modest but statistically significant monotonic trend (Pearson *r* = 0.20, 95% CI –0.10–0.47, *p* = 0.1810; Spearman *r* = 0.46, 95% CI –0.18–0.67, *p* = 0.0016) (Supplementary Fig. 5B). Thus, while time to detection broadly reflected viral load, the assay maintained rapid performance across the full range of *Ct* values observed.

On unextracted lesion swabs, Mpox SHINE achieved a sensitivity of 100% (95% CI 72.2–100.0%) and a specificity of 100% (95% CI 72.2–100.0%) relative to qPCR (Fig. 3B) in this pilot evaluation. We tested eight qPCR-positive and eight qPCR-negative samples in duplicate (32 reactions total, Supplementary Fig. 4). All eight positives were detected, and all eight negatives remained negative. Because matrix-specific controls were not run on-site, thresholds for these analyses were set retrospectively using matched matrix controls containing viral transport media (VTM) and lysis buffer (Fig. 3B, Supplementary Figs. 3D, 5C). These thresholds were informed by prior matrix-development experiments on both the DxHub and a plate reader (Supplementary Fig. 3A–C). Given the limited sample size, these results support a strong proof-of-concept demonstration that Mpox SHINE can detect viral genome from minimally processed skin lesion swabs following ambient temperature chemical lysis, positioning the assay for significant potential in future deployment to decentralized settings.

Time to detection on unextracted samples was slower than on extracted DNA but remained actionable for outbreak response (Fig. 3C). The assay identified positives in an average of 27.9 ± 10.7 min, with 50% detected within 21 min and 100% within 44 min, consistent with control reactions in the same VTM/buffer matrix (Supplementary Fig. 5C). These findings indicate that Mpox SHINE can be applied directly to minimally processed samples while maintaining concordant classification, albeit with delayed kinetics compared to extracted workflows.

## Discussion

This study demonstrates that highly sensitive SHINE-based pathogen detection can be adapted, integrated, and field-validated within the timeline of an active outbreak. More broadly, we show that CRISPR-based diagnostics can be mobilized from genomic sequence to in-country clinical sample validation on outbreak-relevant timescales. In the middle of the 2025 Sierra Leone mpox surge, we went from sequence access to in-country clinical sample testing in less than three months (Supplementary Fig. 6). In practice, the design cycle itself was much faster: assay targets were identified within days of genome release, lyophilized reagents were prepared and validated within weeks, and integration onto a portable isothermal device required only minimal adaptation. With streamlined coordination, these steps could be compressed even further into a matter of days to weeks, underscoring the feasibility of moving from pathogen sequence to field-ready diagnostic tools on outbreak timescales.

Mpox SHINE achieved strong performance on both extracted and unextracted clinical samples. On 56 extracted samples, all positives and negatives were correctly classified, with all positives detected within 37 min and most within 15 min. The same chemistry translated to unextracted lesion swabs processed by chemical lysis, with complete agreement across 16 additional samples. Time to detection on unextracted samples averaged under 30 min, placing actionable results well within the window for clinical decision-making or public health triage. Together, these results establish Mpox SHINE as a portable, rapid, and accurate diagnostic that works not only on purified DNA but also on minimally processed samples, a critical step toward true point-of-care deployment.

The DxHub platform enabled real-time fluorescence monitoring in a portable format, supporting decentralized surveillance. While not a hand-held test nor a sample-to-answer cartridge based system^20,21^, the platform required minimal equipment and hands-on time, making it suitable for rural health facilities. The device’s modular design supports not only Mpox SHINE but also a wide range of isothermal assays, creating a flexible platform for point-of-care testing. Prior SHINE studies demonstrated equipment-free workflows for SARS-CoV-2 and influenza^17,18^, but this work extends the approach into the setting of an ongoing epidemic in West Africa, with assays validated on-site in Sierra Leone. Furthermore, the performance characteristics of Mpox SHINE align with key attributes outlined in WHO diagnostic Target Product Profiles for decentralized testing, including rapid turnaround (<1 h), high sensitivity and specificity, minimal hands-on workflow, and compatibility with minimally processed samples. Together, these features support the feasibility of deploying CRISPR-based diagnostics in decentralized outbreak settings.

Together, these results demonstrate that a CRISPR-based assay can be rapidly designed, integrated into a portable fluorescence platform, and validated in-country during an active outbreak. By advancing from sequence-informed assay design to clinical sample testing at Kenema Government Hospital within a single outbreak window, Mpox SHINE provides a practical model for outbreak-time testing mobilization. While further work is needed to expand simplified sample preparation workflows and assess long-term reagent stability, the performance observed on both extracted and minimally processed samples supports the feasibility of decentralized CRISPR testing in resource-limited clinical settings. More broadly, this study illustrates how established assay chemistry, computational design tools, and deployable instrumentation can be combined into an operational diagnostic pipeline capable of delivering actionable molecular results on outbreak-relevant timescales.

## Limitations

This study has several limitations. First, the clinical sample cohort was modest in size, particularly for the unextracted workflow, which was evaluated on a subset of 16 lesion swab samples. Although all samples were correctly classified relative to qPCR in this pilot evaluation, the resulting confidence intervals are wide. Larger studies across multiple sample collection sites and patient populations will be important to more precisely estimate diagnostic performance.

Second, the current workflow does not represent a fully automated, cartridge-based, sample-to-answer diagnostic. Instead, the SHINE-DxHub system was designed for decentralized surveillance environments such as rural clinics or field laboratories, where infrastructure may be limited but basic sample handling and pipetting are feasible by minimally trained staff. In this context, the combination of short, ambient-temperature lysis, lyophilized reagents, and portable fluorescence detection enables near-point-of-care testing and surveillance with minimal equipment and short turnaround times. Future integration of simplified sample preparation or cartridge-based formats could further streamline the workflow toward fully automated point-of-care deployment.

Third, the assay was optimized for rapid and highly sensitive qualitative detection rather than quantitative viral load measurement. Because the SHINE workflow couples RPA amplification with Cas13-mediated collateral cleavage, fluorescence signals tend to saturate quickly once the target is present. As a result, the assay exhibits a limited dynamic range and is intended for binary viral detection near the limit of detection rather than quantitative measurement.

Finally, while the SHINE chemistry is compatible with lyophilization and has previously been shown to remain stable under extended storage conditions, the Mpox SHINE formulation used in this study was stored at −20 °C during assay development and deployment. Systematic stability testing across storage durations and temperatures, including room temperature, will be important to further define long-term field readiness.

## Methods

### Ethical statement

All research activities complied with relevant ethical regulations and institutional policies and were approved by the appropriate institutional review boards. Research activities at Kenema Government Hospital (KGH) were conducted under approval from the Sierra Leone Ethics and Scientific Review Committee (protocol 002/05/2024). Research activities at the Broad Institute were conducted under approvals from the Harvard Longwood Campus Institutional Review Board (protocols IRB24-0562, IRB24-0563). These studies utilized clinical excess specimens; the secondary use of these biological specimens was approved with a waiver of informed consent in accordance with the aforementioned protocols.

### Mpox SHINE assay designs

#### Guide RNA design

Mpox genomes were retrieved from NCBI (TaxID 10244) with sequence length ≥180,000 bp and collection date ≥2022-01-01 (downloaded 2025-02-07). From these, 50 random genomes each from Clade IIb lineage B.1 (target set), Clade Ia and Clade Ib (non-target sets) were chosen. ADAPT (Activity-informed Design with All-inclusive Patrolling of Targets)^15^ was used to generate candidate crRNA designs by inputting the Mpox IIb-B.1 sequences as the target set. Consensus sequences of Mpox Ia and Ib were computed after alignment using MAFFT via Geneious software and used in ADAPT as non-targets to ensure specificity of crRNA candidates to Mpox IIb B.1. ADAPT crRNA designs were mapped against a larger subset of mpox IIb-B.1, Ia, and Ib sequences (also obtained from NCBI Virus) to select for maximal inclusion to Mpox IIb and minimal predicted detection of Mpox Ia or Ib. A crRNA was considered to detect a sequence if the crRNA mapped to the sequence with ≤ 2 mismatches for on-target sequences or ≤ 3 mismatches for off-target sequences. To validate the assay on the Sierra Leone mpox outbreak strain, the assay was mapped against the first 67 sequences obtained from the active outbreak (Pathoplexus). No mismatches were detected in any of these 67 sequences. The crRNA aligns from nucleotides 156,555 to 156,582 of the mpox genome (NC_063383).

#### Primer design

RPA forward and reverse primers were also designed using ADAPT. ADAPT was run on a multiple sequence alignment of Mpox viral genome sequences obtained from Kenema Government Hospital (KGH), with the following parameters: 35.0% to 65.0% guanine-cytosine (GC) content, max. 3 mismatches between the primer and target, full coverage in 98% of the genomes. The forward and reverse primers align to the mpox genome (NC_063383) from nucleotides 156,525 to 156,554 and 156,583 to 156,612, respectively, with 0 mismatches detected in any of the input sequences. Forward RPA primers were ordered with and without a T7 promoter sequence (5’-GAAATTAATACGACTCACTATAGGG-3’) appended upstream. Both forward and reverse RPA primers and crRNAs were purchased from Integrated DNA Technologies. A synthetic DNA target sequence was ordered as a double-stranded DNA gene fragment (dsDNA) from Twist Biosciences for use as a positive control and during assay development.

### Sequence alignment and percent identity analysis

Full-length reference genomes were obtained from NCBI RefSeq for Vaccinia (NC_006998.1), Cowpox (NC_003663.2) and Camelpox (AY009089.1), while clades Ia, Ib, IIa and IIb mpox genomes (including sequences from the 2025 Sierra Leone outbreak) were sourced from Pathoplexus^16^. To assess conservation of the SHINE assay amplicon (nt 156,525 to 156,612 with respect to NC_063383) across mpox clades and other Orthopoxviruses, we performed multiple sequence alignment using MAFFT v7.490^22,23^ implemented in Geneious Prime (Biomatters Ltd.) with default parameters (gap opening penalty = 1.53; offset value = 0.123). Pairwise percent identity values were then calculated in Geneious as the proportion of identical nucleotides across the alignment length, with mismatches and gaps counted as differences. Amplicon absence from clade I (Ia/Ib) lineages was confirmed by the presence of characteristic deletions spanning this locus after alignment.

### Mpox SHINE master mix preparation

SHINE reactions were performed using the following conditions: 1 × Lyo buffer (20 mM HEPES pH 8.0, 150 mM D-Mannitol and 5 % w/v Sucrose), 45 nM LwaCas13a protein (Genscript, Z03486-100) resuspended in 1 × storage buffer (50 mM Tris-HCl pH 7.5, 600 mM NaCl, 2 mM DTT, and 5% Glycerol), 62.5 nM 6-U quenched FAM reporter (IDT), 2 mM of each ribonucleoside triphosphate (rNTP) (NEB, N0466S), 1 U/μL murine RNase inhibitor (NEB, M0314L), 1 U/μL NextGen T7 RNA polymerase (Biosearch Technologies, 30223-1), 0.1 U/μL RNase H (NEB, M0297L), 240-360 nM Forward RPA primer (120–180 nM Fwd primer with T7 promoter and 120–180 nM Forward primer without T7 promoter), 240–360 nM Reverse RPA primer (IDT), and 22.5 nM crRNA (IDT). TwistAmp basic kit RPA pellets (TwistDx, TABAS03KIT) were used at a ratio of 1 pellet per 100 μL of master mix. The final master mix was aliquoted to single-reaction volumes in individual 0.2 mL tubes, flash frozen in liquid nitrogen, and immediately set to lyophilize in a Labconco FreeZone 70040 4.5 L Freeze Dryer overnight at −50 °C and a vacuum pressure of <0.4 mbar. The following day, freeze-dried Mpox SHINE pellets were either used immediately or stored at −20 °C for later use.

### Mpox SHINE plate reader fluorescence data collection

Reaction pellets were rehydrated with a resuspension buffer (RB at 1x: 60 mM KCl, 3.5% w/v PEG-8000, and 25 mM Magnesium Acetate), leaving volume space to add synthetic gene target or water as a negative control up to 30 μL final volume. Fluorescence kinetics were measured on a Biotek Cytation 5 plate reader (Agilent, USA) with excitation at 485 nm and emission at 520 nm at 38 °C every 20 s for up to 90 mins.

### Mpox SHINE DxHub fluorescence data collection

For DxHub reactions, fluorescence signals were measured on a commercial DxHub device (DxLab Inc., Somerville, Massachusetts, United States; manufactured under contract by Axxin, Eaglemont, Australia) at 38 °C every 20 s for up to 60 mins. For controls and extracted clinical samples, the DxHub was operated at 50% of the device’s maximum FAM signal detection sensitivity; for unextracted samples, a 10% FAM sensitivity was used. DxHub was powered using an AC/DC power adapter provided with the device or a portable power bank.

### Analytical LOD determination

To determine the analytical limit of detection (LOD), a 10-fold serial dilution of synthetic Mpox gene targets was prepared in nuclease-free water, ranging from 10^6^ to 10^0^ copies/µL. Each concentration was tested in technical replicates. The LOD was determined based on the detection frequency across these replicates using the criteria described in the Statistics & Reproducibility section.

### Production and transport of Mpox SHINE pellets for clinical sample testing in Sierra Leone

SHINE reaction pellets were prepared at the Broad Institute, lyophilized overnight (LabConco Freezone 4.5 L freeze dryer), and packed in sets of 24 pellets in an aluminum vacuum-sealed pack to minimize fluorescence quenching and with a silica gel desiccant included inside to minimize moisture buildup. Twenty-five vacuum packs of over 500 individual lyophilized SHINE pellets were prepared for transit to Kenema, Sierra Leone. All cold items were packed in a Credo DURACUBE Reusable Cold Chain Shipper, which maintained a steady temperature of −20 °C during travel.

### Clinical samples and RADI FAST qPCR

Clinical samples were collected in the form of skin-lesion swabs stored in viral transport media (VTM). Clinical negatives were obtained from close contacts of suspected cases during the outbreak period. Clinical samples were extracted by lab personnel at the KGH molecular laboratory in a BSL-3 facility, using the QIAamp DNA Micro kit (Qiagen, 56304) using the protocol for Isolation of Genomic DNA from Tissues. Extractions were performed with an input volume of 400 μL of sample (Viral transfer media with swabbed lesion material) and an elution volume of 200 μL using water. Extracted DNA was aliquoted for different assays and stored at −20 °C.

To establish a reference standard, all samples underwent repeat qPCR testing using the RADI FAST Mpox Detection Kit (IVD) (KH Medical, RV015) on a Roche LightCycler 480. In accordance with the manufacturer’s protocol, samples were categorized based on cycle threshold (*Ct*) values, where a *Ct* ≤ 40 was considered positive. Cycling conditions were as follows: hold at 95 °C for 20 s, cycle at 95 °C for 2 s and 60 °C for 5 s for 45 cycles. Real-time qPCR was run on a Roche LightCycler480. Data was analyzed using the LightCycler 480 II software version 1.5.1.62 SP3.

### Mpox SHINE testing for extracted clinical samples

Lyophilized SHINE pellets were stored at −20 °C; only the number required for a run (up to eight) were removed at a time. In a target-free, ventilated hood, each pellet was rehydrated with 15 µL of 2 × Resuspension Buffer (120 mM KCl, 7.0% PEG-8000, 50 mM magnesium acetate), vortexed until fully dissolved, and briefly centrifuged. The resuspension was transferred to an individual dome-capped PCR tube. In a separate pre-amplification, target-handling hood, 15 µL of extracted clinical sample was added to the tube, mixed, and briefly spun down. Tubes were loaded into the DxHub and incubated at 38 °C for a 60-min Mpox SHINE run, with fluorescence signals measured every 20 s.

### Assessment of compatibility of lysis buffers with the mpox SHINE reaction

Initial testing was done on the plate reader to determine mpox SHINE reaction compatibility with the expected sample matrix (VTM) in combination with two different lysis buffers. Buffer 1, aka “Saliva Lysis Buffer”: 12.5 mM TCEP, 5 mM EDTA, 1% Pluronic, 55 mM NaOH^24^; Buffer 2: 5x Fast-Amp Viral and Cell Lysis Solution (IntactGenomics, 4631) was combined with VTM and included in mpox SHINE reactions at different volume proportions to determine the effect on sensitivity and reaction kinetics (Supplementary Fig. 3A–C). Buffer 1 was chosen based on the observed reaction performance and tested on the DxHub at 30% volume (Supplementary Fig. 3D, Fig. 3B - left side).

### Chemical lysis of unextracted mpox samples

Prior to testing of unextracted clinical samples, Buffer 1 was prepared in a target-free hood and aliquoted into PCR tubes (6 µL per tube; one tube per sample). Tubes were transferred into the BSL-3 laboratory, where 24 µL of unextracted clinical sample was added to each tube. After pipette mixing and a brief centrifugation, tubes were incubated at room temperature for ≥5 min to achieve chemical lysis and viral inactivation. The lysed samples were then ready for addition to SHINE reactions.

### Mpox SHINE testing for unextracted clinical samples

To minimize any potential autofluorescence and inhibitory interactions from unextracted matrices, pellets were rehydrated with a magnesium-only buffer (no PEG/KCl), formulated so that after sample addition, the final Mg²⁺ concentration was 30 mM. In a target-free hood, each pellet was rehydrated with 21 µL, vortexed to fully dissolve, and transferred to a PCR tube. Tubes were then taken into the BSL-3 laboratory, where 9 µL of chemically lysed clinical sample was added, mixed, and briefly centrifuged. The assembled reactions were returned to the main lab and run on the DxHub. Because VTM raised baseline fluorescence on the device, the DxHub FAM sensitivity was set to 10% to prevent early saturation and improve interpretation.

### Statistics and reproducibility

#### Study design and sample size rationale

No statistical method was used to predetermine sample size. The clinical sample size (*n* = 56) was determined by the availability of excess lesion swabs collected at Kenema Government Hospital during the active 2025 Sierra Leone Mpox clade IIb outbreak window. For the pilot evaluation of unextracted samples, a subset of 16 samples (8 qPCR-positive and 8 qPCR-negative) was selected to provide a proof-of-concept for extraction-free workflows. While ideally negative controls would include specimens from patients with other skin–lesion–causing illnesses or infections with related orthopox viruses, such samples were not available during the study period.

#### Data exclusion

A total of 57 clinical samples were initially provided. One sample was excluded from the final analysis because it returned a borderline qPCR cycle threshold (*Ct*) of 40, which was deemed of insufficient reliability for a definitive positive reference. No other data were excluded from the analyses.

#### Replication and reproducibility

All attempts at replication were successful. Reagents during analytical testing were validated across two different platforms (Biotek Cytation 5 and DxHub) to ensure platform-independent reproducibility. Each clinical sample was tested in triplicate to provide statistical rigor, mitigate stochastic effects at low template concentrations, and enable application of replicate-based interpretation rules. For unextracted clinical samples, duplicate reactions were run.

#### Randomization and blinding

The experiments were not randomized, as clinical samples were categorized based on a reference qPCR standard to evaluate diagnostic performance. The investigators were not blinded to allocation during experiments and outcome assessment.

#### Statistical analysis and result calling

Fluorescence data from both plate reader and DxHub assays were analyzed using a uniform approach. A positivity threshold was set at the mean endpoint (time = 60 mins) fluorescence of negative template control (NTC) reactions plus 10 standard deviations to minimize false-positive calls. At the sample level, results for extracted samples were called positive if at least 2 of 3 replicates exceeded the threshold. For unextracted samples, a result was called positive if both (2 of 2) replicates exceeded the threshold. Sensitivity (true positive rate) was defined as the proportion of qPCR-positive samples correctly identified as positive by SHINE, and specificity (true negative rate) as the proportion of qPCR-negative samples correctly identified as negative. Point estimates of sensitivity and specificity were calculated in GraphPad Prism (v10.2) with 95% one-sided confidence intervals using the Wilson score method, appropriate for small sample sizes and binomial outcomes. All plots and statistical summaries were generated in GraphPad Prism, and results were confirmed by manual cross-checking of replicate data.

### Declaration of generative AI and AI-assisted technologies in the writing process

During the preparation of this work, the authors used AI-assisted technologies and large language models to refine phrasing, improve overall grammatical structure, and screen for detailed technical formatting and consistency errors throughout the manuscript and supplementary materials. After using these tools, the authors reviewed, edited, and modified the resulting content as needed, and take full responsibility for the accuracy and scientific integrity of the final peer-reviewed publication.

### Reporting summary

Further information on research design is available in the Nature Portfolio Reporting Summary linked to this article.

## Supplementary information

Supplementary Information

Description of Additional Supplementary Information

Supplementary Data 1

Supplementary Data 2

Reporting Summary

Transparent Peer Review file

## Source data

Source Data

## Data Availability

All data supporting the findings of this study are available in the main text or the Supplementary Materials. Source data are provided with this paper. The outbreak-relevant Mpox virus Clade IIb genome sequences used for assay design were sourced from the Pathoplexus database. These public datasets, along with the standard NCBI reference sequence assemblies utilized in this study for alignment and conservation analysis, are permanently available at the following accession codes and hyperlinks: **Outbreak study genomes (Pathoplexus):** ● PP_002XLGK.1 [https://pathoplexus.org/seq/PP_002XLGK.1] (Mpox virus isolate G-29022-1 from Sierra Leone) ● PP_002XLHG.1 [https://pathoplexus.org/seq/PP_002XLHG.1] (Mpox virus isolate G-29023-1 from Sierra Leone) ● PP_002XKVT.1 [https://pathoplexus.org/seq/PP_002XKVT.1] (Mpox virus isolate G-28995-1 from Sierra Leone) ● PP_002XKUV.1 [https://pathoplexus.org/seq/PP_002XKUV.1] (Mpox virus isolate G-28994-1 from Sierra Leone) ● PP_002XL0H.1 [https://pathoplexus.org/seq/PP_002XL0H.1] (Mpox virus isolate G-29004-1 from Sierra Leone) ● PP_002XKWR.1 [https://pathoplexus.org/seq/PP_002XKWR.1] (Mpox virus isolate G-29000-1 from Sierra Leone) ● PP_002XKXP.1 [https://pathoplexus.org/seq/PP_002XKXP.1] (Mpox virus isolate G-29001-1 from Sierra Leone) ● PP_002XKSZ.1 [https://pathoplexus.org/seq/PP_002XKSZ.1] (Mpox virus isolate G-28997-1 from Sierra Leone) ● PP_002XKTX.1 [https://pathoplexus.org/seq/PP_002XKTX.1] (Mpox virus isolate G-28998-1 from Sierra Leone) ● PP_002XLN6.1 [https://pathoplexus.org/seq/PP_002XLN6.1] (Mpox virus isolate G-28999-1 from Sierra Leone) ● PP_002XKYM.1 [https://pathoplexus.org/seq/PP_002XKYM.1] (Mpox virus isolate G-29002-1 from Sierra Leone) ● PP_002XKZK.1 [https://pathoplexus.org/seq/PP_002XKZK.1] (Mpox virus isolate G-29003-1 from Sierra Leone) ● PP_002XL1F.1 [https://pathoplexus.org/seq/PP_002XL1F.1] (Mpox virus isolate G-29005-1 from Sierra Leone) ● PP_002XL2D.1 [https://pathoplexus.org/seq/PP_002XL2D.1] (Mpox virus isolate G-29007-1 from Sierra Leone) ● PP_002XLBV.1 [https://pathoplexus.org/seq/PP_002XLBV.1] (Mpox virus isolate G-29008-1 from Sierra Leone) ● PP_002XL49.1 [https://pathoplexus.org/seq/PP_002XL49.1] (Mpox virus isolate G-29011-1 from Sierra Leone) ● PP_002XL57.1 [https://pathoplexus.org/seq/PP_002XL57.1] (Mpox virus isolate G-29012-1 from Sierra Leone) ● PP_002XL65.1 [https://pathoplexus.org/seq/PP_002XL65.1] (Mpox virus isolate G-29013-1 from Sierra Leone) ● PP_002XL3B.1 [https://pathoplexus.org/seq/PP_002XL3B.1] (Mpox virus isolate G-29010-1 from Sierra Leone) ● PP_002XL73.1 [https://pathoplexus.org/seq/PP_002XL73.1] (Mpox virus isolate G-29014-1 from Sierra Leone) ● PP_002XLDR.1 [https://pathoplexus.org/seq/PP_002XLDR.1] (Mpox virus isolate G-29015-1 from Sierra Leone) ● PP_002XLEP.1 [https://pathoplexus.org/seq/PP_002XLEP.1] (Mpox virus isolate G-29016-1 from Sierra Leone) ● PP_002XLFM.1 [https://pathoplexus.org/seq/PP_002XLFM.1] (Mpox virus isolate G-29017-1 from Sierra Leone) ● PP_002XL81.1 [https://pathoplexus.org/seq/PP_002XL81.1] (Mpox virus isolate G-29019-1 from Sierra Leone) ● PP_002XL9Z.1 [https://pathoplexus.org/seq/PP_002XL9Z.1] (Mpox virus isolate G-29020-1 from Sierra Leone) ● PP_002XLAX.1 [https://pathoplexus.org/seq/PP_002XLAX.1] (Mpox virus isolate G-29021-1 from Sierra Leone) ● PP_002XLJE.1 [https://pathoplexus.org/seq/PP_002XLJE.1] (Mpox virus isolate G-29025-1 from Sierra Leone) ● PP_002XLKC.1 [https://pathoplexus.org/seq/PP_002XLKC.1] (Mpox virus isolate G-29027-1 from Sierra Leone) ● PP_002XLLA.1 [https://pathoplexus.org/seq/PP_002XLLA.1] (Mpox virus isolate G-29028-1 from Sierra Leone) ● PP_002XLM8.1 [https://pathoplexus.org/seq/PP_002XLM8.1] (Mpox virus isolate G-29029-1 from Sierra Leone) ● PP_0031UPN.1 [https://pathoplexus.org/seq/PP_0031UPN.1] (Mpox virus isolate G-29030-1 from Sierra Leone) ● PP_0031UQL.1 [https://pathoplexus.org/seq/PP_0031UQL.1] (Mpox virus isolate G-29034-1 from Sierra Leone) ● PP_0031URJ.1 [https://pathoplexus.org/seq/PP_0031URJ.1] (Mpox virus isolate G-29036-1 from Sierra Leone) ● PP_0031USG.1 [https://pathoplexus.org/seq/PP_0031USG.1] (Mpox virus isolate G-29038-1 from Sierra Leone) ● PP_0031UTE.1 [https://pathoplexus.org/seq/PP_0031UTE.1] (Mpox virus isolate G-29040-1 from Sierra Leone) ● PP_0031UUC.1 [https://pathoplexus.org/seq/PP_0031UUC.1] (Mpox virus isolate G-29041-1 from Sierra Leone) ● PP_0031UVA.1 [https://pathoplexus.org/seq/PP_0031UVA.1] (Mpox virus isolate G-29042-1 from Sierra Leone) ● PP_0031UW8.1 [https://pathoplexus.org/seq/PP_0031UW8.1] (Mpox virus isolate G-29043-1 from Sierra Leone) ● PP_0031UX6.1 [https://pathoplexus.org/seq/PP_0031UX6.1] (Mpox virus isolate G-29044-1 from Sierra Leone) ● PP_0031UY4.1 [https://pathoplexus.org/seq/PP_0031UY4.1] (Mpox virus isolate G-29049-1 from Sierra Leone) ● PP_0031UZ2.1 [https://pathoplexus.org/seq/PP_0031UZ2.1] (Mpox virus isolate G-29050-1 from Sierra Leone) ● PP_0031V00.1 [https://pathoplexus.org/seq/PP_0031V00.1] (Mpox virus isolate G-29051-1 from Sierra Leone) ● PP_0031V1Y.1 [https://pathoplexus.org/seq/PP_0031V1Y.1] (Mpox virus isolate G-29052-1 from Sierra Leone) ● PP_0031V2W.1 [https://pathoplexus.org/seq/PP_0031V2W.1] (Mpox virus isolate G-29053-1 from Sierra Leone) ● PP_0031V3U.1 [https://pathoplexus.org/seq/PP_0031V3U.1] (Mpox virus isolate G-29054-1 from Sierra Leone) ● PP_0031V4S.1 [https://pathoplexus.org/seq/PP_0031V4S.1] (Mpox virus isolate G-29058-1 from Sierra Leone) ● PP_0031V50.1 [https://pathoplexus.org/seq/PP_0031V50.1] (Mpox virus isolate G-29059-1 from Sierra Leone) ● PP_0031V7L.1 [https://pathoplexus.org/seq/PP_0031V7L.1] (Mpox virus isolate G-29065-1 from Sierra Leone) ● PP_0031V8J.1 [https://pathoplexus.org/seq/PP_0031V8J.1] (Mpox virus isolate G-29066-1 from Sierra Leone) ● PP_0031V6N.1 [https://pathoplexus.org/seq/PP_0031V6N.1] (Mpox virus isolate G-29061-1 from Sierra Leone) ● PP_0031V9G.1 [https://pathoplexus.org/seq/PP_0031V9G.1] (Mpox virus isolate G-29070-1 from Sierra Leone) ● PP_0031VAG.1 [https://pathoplexus.org/seq/PP_0031VAE.1] (Mpox virus isolate G-29071-1 from Sierra Leone) ● PP_0031VD8.1 [https://pathoplexus.org/seq/PP_0031VD8.1] (Mpox virus isolate G-29110-1 from Sierra Leone) ● PP_0031VBC.1 [https://pathoplexus.org/seq/PP_0031VBC.1] (Mpox virus isolate G-29102-1 from Sierra Leone) ● PP_0031VE6.1 [https://pathoplexus.org/seq/PP_0031VE6.1] (Mpox virus isolate G-29111-1 from Sierra Leone) ● PP_002XLCT.1 [https://pathoplexus.org/seq/PP_002XLCT.1] (Mpox virus isolate G-29009-1 from Sierra Leone) ● PP_0031VCA.1 [https://pathoplexus.org/seq/PP_0031VCA.1] (Mpox virus isolate G-29106-1 from Sierra Leone) ● PP_0031VF4.1 [https://pathoplexus.org/seq/PP_0031VF4.1] (Mpox virus isolate G-29125-1 from Sierra Leone) ● PP_0031VG2.1 [https://pathoplexus.org/seq/PP_0031VG2.1] (Mpox virus isolate G-29126-10) from Sierra Leone) ● PP_0031VHZ.1 [https://pathoplexus.org/seq/PP_0031VHZ.1] (Mpox virus isolate G-29127-1 from Sierra Leone) ● PP_0031VJX.1 [https://pathoplexus.org/seq/PP_0031VJX.1] (Mpox virus isolate G-29128-1 from Sierra Leone) ● PP_0031VKV.1 [https://pathoplexus.org/seq/PP_0031VKV.1] (Mpox virus isolate G-29129-1 from Sierra Leone) **Reference genomes (NCBI GenBank):** ● NC_063383 [https://www.ncbi.nlm.nih.gov/nuccore/NC_063383] (Mpox virus Clade IIb reference genome) ● NC_006998.1 [https://www.ncbi.nlm.nih.gov/nuccore/NC_006998.1] (Vaccinia virus reference genome) ● NC_003663.2 [https://www.ncbi.nlm.nih.gov/nuccore/NC_003663.2] (Cowpox virus reference genome) ● AY009089.1 [https://www.ncbi.nlm.nih.gov/nuccore/AY009089.1] (Camelpox virus reference genome) Source data are provided with this paper.
