# Supplementary material for: Rapid development and field evaluation of a portable CRISPR-based assay for Mpox during the 2025 Sierra Leone outbreak
Source: Nat Commun. 2026 Jun 6;17:7241. doi: 10.1038/s41467-026-74034-8 (PMC13396451; doi:10.1038/s41467-026-74034-8)
Supplement: Supplementary file 1 — Supplementary Information [file 41467_2026_74034_MOESM1_ESM.pdf]

Supplementary Table 1. Assay designs for Mpox SHINE

Component	Sequence
Forward primer (5'-3') with 5’ T7 promoter	GAAATTAATACGACTCACTATAGGGGAATTTGCCAAGG<br>TCAGATGTAGAGTTGGT
Forward primer (5'-3') without 5’ T7 promoter	GAATTTGCCAAGGTCAGATGTAGAGTTGGT
Reverse primer (5'-3')	AATTGGGGATTGCGGTATATGTATGAGGTG
CRISPR RNA (5'-3')	GGCAGUGUUCACAUUUUGUGUUAUCACU
Mpox dsDNA gene fragment (5’-3’)	GAAATTAATACGACTCACTATAGGGAGATTTTGTTATTG<br>TAGTATGATAATATCAAAAAGATGGATATAAAGAATTTA<br>CTGACTACATGTACTATTTTACATTACTACATTGGCTAC<br>GGCATATATACCTATTTCGTCACTTCCACACGCTCCGGT<br>AAACGGGTGTCATGTGACGAGGGAGAATCTTGATAAG<br>AGGCATAATCAATGTTGTAATCCGATGTCCACCTGGAG<br>AATTTGCCAAGGTCAGATGTAGAGTTGGTAGTGATAAC<br>ACAAAATGTGAACACTGCCCACCTCATACATATACCGC<br>AATCCCCAATTATTCTAATAGATGTCATCAATGTAGAAA<br>ATGCCCAACAGGATCATTTGATAAGGTAAAGTGTACCG<br>GAACACAGAACAAATGTTCGTGTCATCCTGGTTGGTAT<br>ACGCTACTGATTCTTCACAGACTGAAGATTGTCGAGAT<br>TTGTGTACCAAAAAAGGAGATGTCCATGCGGATACTTT<br>GGTGGAATAGATGAAGGAAATCCTATT
Fluorescent Probe (5’-3’)	/56-FAM/rUrUrUrUrUrU/3IABkFQ/

Supplementary Table 2. Comparison between Mpox SHINE and two alternative CRISPR-based point of care assay platforms.

	Mpox SHINE	Thermally regulated asynchronous CRISPR-enhanced (TRACE) (1)	Streamlined CRISPR On Pod Evaluation platform (SCOPE) (2)
Analytical Performance	Detection down to ~1 copy/µL; high concordance with clinical qPCR	~2.5 copies/test	~0.5 copies/µL of reaction
Time to result	~11 min (unextracted) to ~28 min (extracted)	~20 min (including lateral flow readout)	~10–15 min incubation with endpoint readout
Sample preparation requirements	~5 min ambient lysis of skin lesion swab; extraction-free compatible	~5 min temperature-agnostic viral lysis	~2 min viral lysis at 80°C
Reagents	Lyophilized reagents	Lyophilized reagents	Lyophilized reagents
Readout modality	Real-time fluorescence via portable DxHub device	Cas13 Fluorescence or lateral flow strip	Fluorescence via integrated “CRISPRPod” vestpocket device
Operational readiness and availability	Compatible with commercially available device (DxHub)	Prototype POC workflow; no standardized commercial device	Prototype integrated device (CRISPRPod); not commercially available
Intended Use Context	Decentralized clinical testing; outbreak deployment	Point-of-care testing	Point-of-care testing in primary care settings

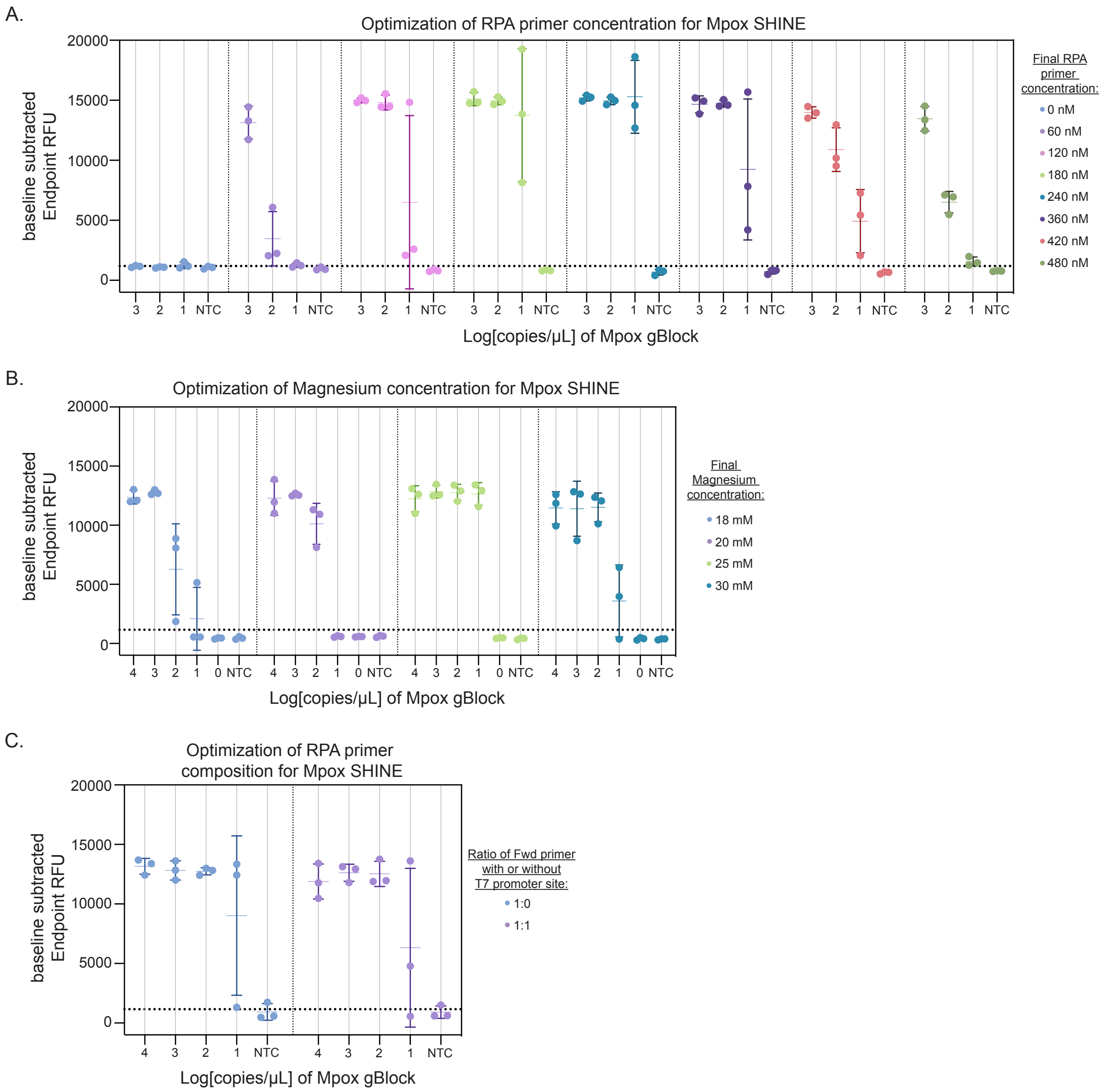

Supplementary Figure 1. Mpox SHINE optimization on plate reader.
(A) Primer concentration titration. Forward and reverse RPA primers tested at the indicated final concentrations (0–480 nM each) against a log dilution of synthetic target (log4 – log0 copies/µL in the reaction) and no-template controls (NTC). 240 nM or 360 nM primer was used for subsequent experiments.
(B) Magnesium titration. Final Mg^{2} (magnesium acetate) was varied (18–30 mM) under the same target series. 25 mM Mg2+ was used for all subsequent experiments.
(C) Forward-primer composition. Comparison of a 1:0 mix (100% primer with T7 promoter) versus a 1:1 mix (50% primer with T7 promoter and 50% primer without T7 promoter); reverse primer and other components held constant. A 1:1 mix was used in all subsequent experiments.
For all panels, points are individual replicates and error bars show mean ± SD of endpoint fluorescence (baseline-subtracted RFU at 60 min; baseline = mean RFU from 1–4 min). The horizontal dashed line marks the fixed RFU calling threshold, defined explicitly as the mean endpoint fluorescence of the no-template controls plus 10 standard deviations (1,644.9 RFU). Reactions were run at 38 °C. NTCs remained below threshold across conditions.

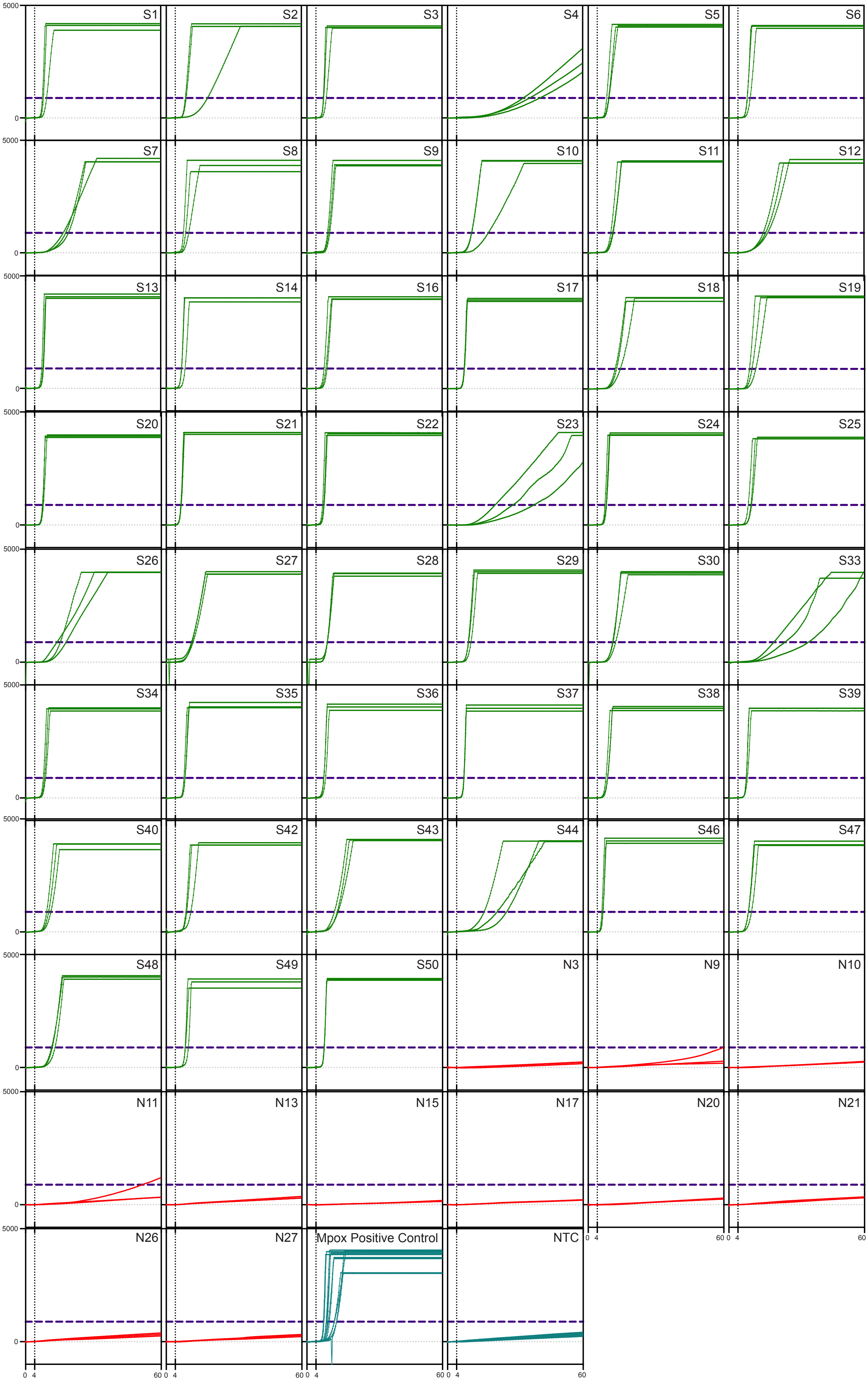

Supplementary Figure 2. Individual fluorescence vs time traces for Mpox SHINE extracted clinical sample tests. Baseline-subtracted FAM fluorescence over time for individual clinical specimens run in triplicate at 38 °C (one panel per specimen). Green traces = qPCR-positive samples; red traces = qPCR-negative samples. The horizontal dashed line marks the fixed RFU threshold. Last two panels in the final row include positive controls (10^3 copies/µL gBlock) and no-template controls (NTCs). X-axes all represent Time (minutes) and Y-axes represent Flourescence in relative fluorescence units (RFU).

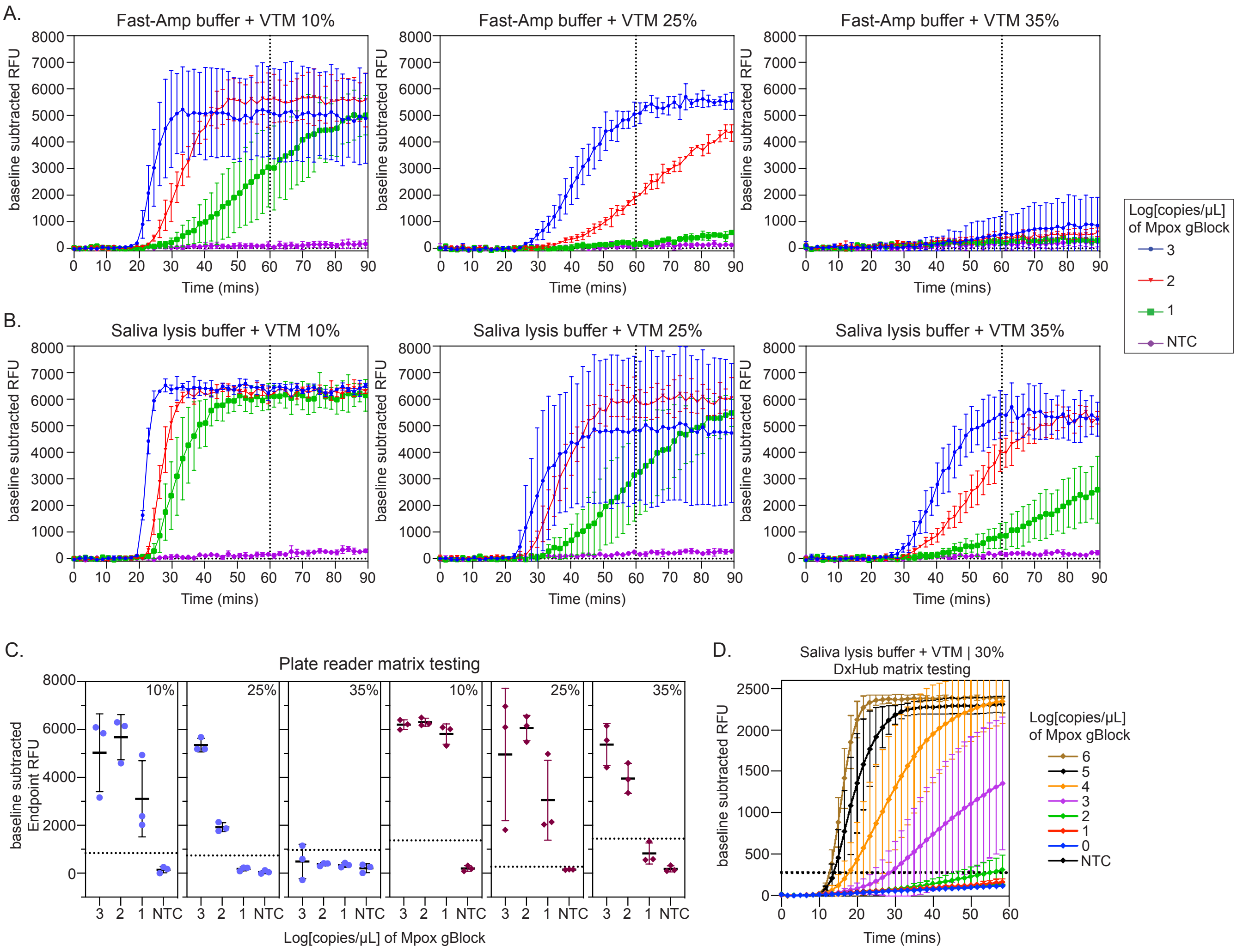

Supplementary Figure 3. Mpox SHINE matrix testing on the plate reader.
(A–B) Real-time FAM fluorescence (baseline-subtracted RFU) for contrived Mpox synthetic target at three input levels (10^3, 10^2, 10^1 copies/µL in the reaction; blue, red, green) and NTCs (magenta) in viral transport medium (VTM) mixed with either FastAmp lysis buffer (IntactGenomics, cat# 4631) (A) or Saliva Lysis Buffer (3) (B). Each panel shows three final matrix fractions per reaction (10%, 25%, 35% VTM+lysis buffer). Lines/points denote the mean; error bars indicate SD across technical replicates. The vertical dashed line marks the 60-min decision time. n = 3 independent technical replicates per condition for these plate reader assays.

(C) Endpoint fluorescence (baseline-subtracted RFU) from the same plate reader experiments shown in panels A and B, summarized by condition (mean + 10 SD, n = 3 technical replicates per condition). Horizontal lines denote thresholds calculated per condition as NTC mean + 10 SD.

(D) Real-time FAM fluorescence measured on the DxHub (baseline-subtracted RFU) for contrived Mpox synthetic target at six input levels (10^6 to 10^0 copies/µL in the reaction) and NTCs in viral transport medium (VTM) mixed with saliva lysis buffer, included in the reaction at 30% volume (9 µL matrix per 30 µL reaction). Replicate sample sizes for this on-device dataset match the primary control analysis detailed in Figure 3b: 10^0-10^1 copies/µL (n = 2), 10^2 copies/µL (n = 7), 10^3 copies/µL (n = 8), 10^4–10^5 copies/µL (n = 6), 10^6 copies/µL (n = 2), and NTCs (n = 8). DxHub FAM sensitivity set to 10% for reactions with matrix.

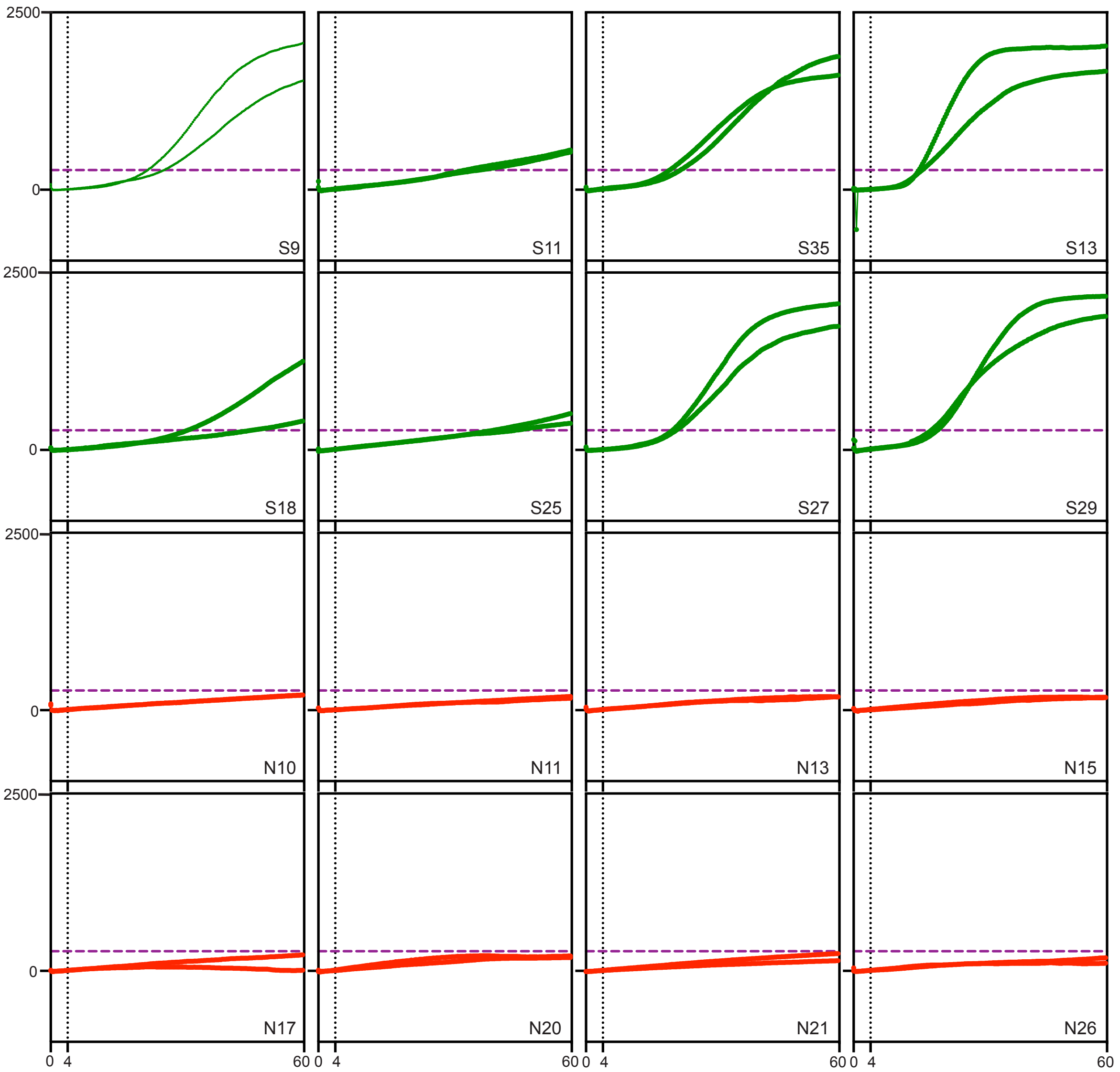

Supplementary Figure 4. Individual fluorescence vs time traces for Mpox SHINE unextracted clinical sample tests. Baseline-subtracted FAM fluorescence over time for individual clinical specimens run in triplicate at 38 °C (one panel per specimen). Green traces = qPCR-positive samples; red traces = qPCR-negative samples. The horizontal dashed line marks the fixed RFU threshold. X-axes all represent Time (minutes) and Y-axes represent Flourescence in relative fluorescence units (RFU).

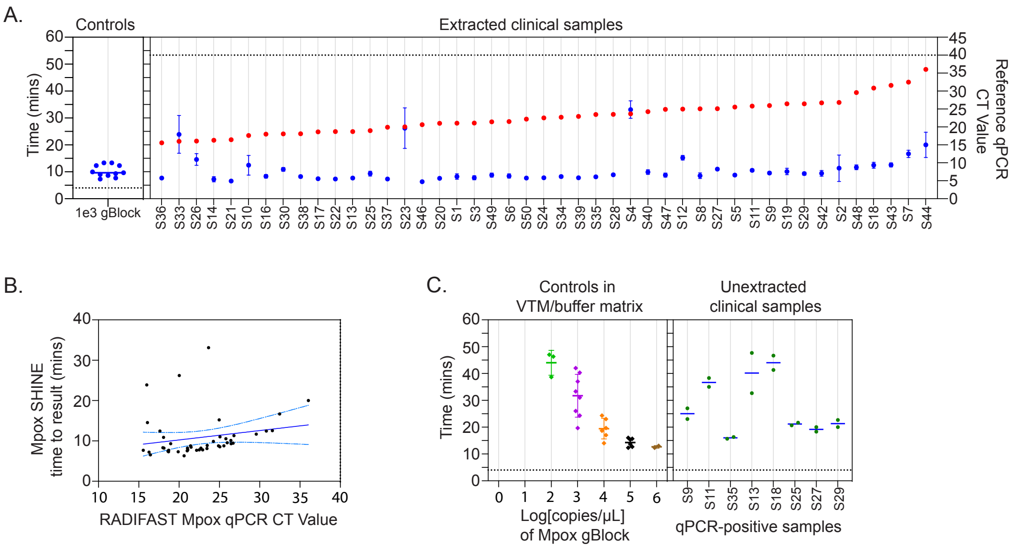

Supplementary Figure 5. Relationship between qPCR Ct values and SHINE time-to-detection.
(A) Time to detection (left y-axis) for controls and Qiagen-extracted clinical samples is plotted in minutes. Control samples (Mpox synthetic target at 1e3 copies/μL, blue symbols, n=11) are shown on the left. qPCR-positive clinical samples are shown on the right (blue circles, n=45 positive samples), and the corresponding qPCR Ct values for each sample are shown as red circles (right y-axis). For each clinical sample, the mean ± SD across 3 technical replicates is plotted, and samples in this figure are plotted in CT value order (right y-axis) from smallest to largest left to right.
(B) Scatterplot of Mpox SHINE time to result values versus corresponding RADIFAST qPCR CT values for extracted clinical samples (n=45). Solid line shows linear regression fit (R squared = 0.0412) with 95% confidence bands (dashed lines). Pearson correlation: Pearson r = 0.20, 95% CI −0.10–0.47, p = 0.1810, not significant. Spearman correlation: ρ = 0.46, 95% CI 0.18–0.67, p = 0.0016, mildly significant.
(C) Time to result values for controls and unextracted clinical samples. Control reactions in VTM + saliva lysis buffer matrix is shown on the left with a 10-fold dilution series of Mpox synthetic target (10^6–10^0 copies/μL of reaction; precise sample sizes per dilution are: 10^0–10^1 copies/μL (n = 2), 10^2 copies/μL (n = 7), 10^3 copies/μL (n = 8), 10^4–10^5 copies/μL (n = 6), 10^6 copies/μL (n = 2), and NTCs (n = 8) (see Figure 3b for replicate details)). qPCR-positive unextracted clinical samples are shown on the right (blue circles, n=8 positive samples). For each clinical sample, the mean across 2 technical replicates is shown.

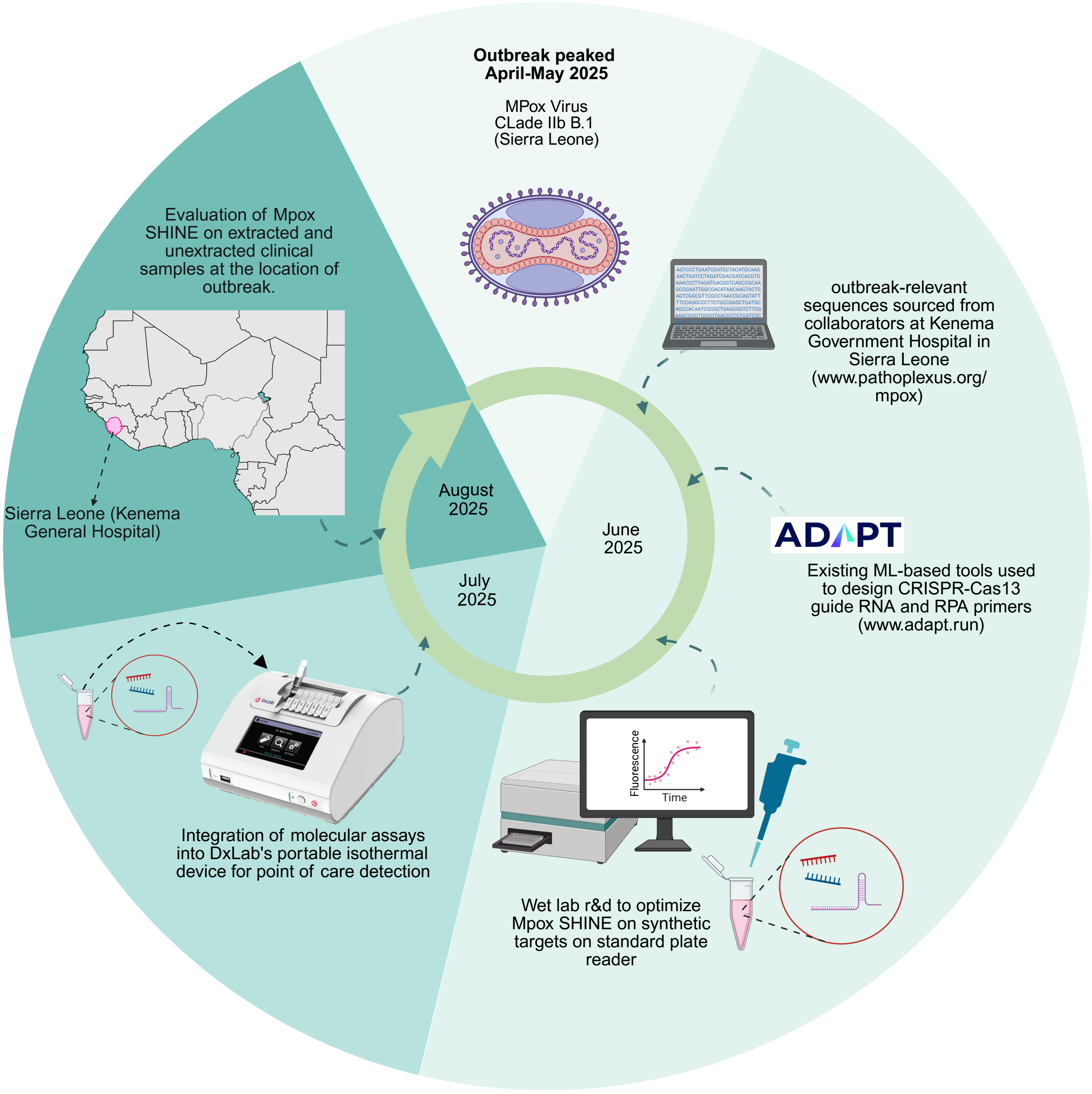

Supplementary Figure 6. Timeline and workflow of assay design, development, device-integration, and in-country validation on clinical samples, in response to the Mpox Sierra Leone 2025 outbreak. Figure made in Biorender. Created in BioRender. Gopal, N. (2026) https://BioRender.com/3phnjcl

Supplementary References

(1) Z. Huang, Y. Dong, Y. Yang, X. Han, F. Wang, C. J. Lyon, S. Ding, Y. Peng, G. Zhang, C. Hu, H. Huang, L. Yang, G. Zhao, X.-Y. Fan, S. Lu, T. Hu, J. Wang, Thermally programmed one-pot CRISPR assay for on-site pandemic surveillance. *Nat Commun* 16, 10286 (2025).
(2) Y. Wang, Y. Tang, Y. Chen, G. Yu, X. Zhang, L. Yang, C. Zhao, P. Wang, S. Gao, Ultrasensitive one-pot detection of monkeypox virus with RPA and CRISPR in a sucrose-aided multiphase aqueous system. *Microbiol Spectr* 12, e02267-23.
(3) Z. Li, A. Sinha, Y. Zhang, N. Tanner, H.-T. Cheng, P. Premsrirut, C. K. S. Carlow, Extraction-free LAMP assays for generic detection of Old World Orthopoxviruses and specific detection of Mpox virus. *Sci Rep* 13, 21093 (2023).
