# Supplementary material for: Rapid development and field evaluation of a portable CRISPR-based assay for Mpox during the 2025 Sierra Leone outbreak
Source: Nat Commun. 2026 Jun 6;17:7241. doi: 10.1038/s41467-026-74034-8 (PMC13396451; doi:10.1038/s41467-026-74034-8)
Supplement: Supplementary file 2 — Description of Additional Supplementary Information [file 41467_2026_74034_MOESM2_ESM.pdf]

Description of Additional Supplementary Files

File Name: Supplementary Data 1
Description: Clinical sample cohort and SHINE validation results.

File Name: Supplementary Data 2
Description: Mpox clade IIb accession numbers via Pathoplexus.
