# Supplementary material for: Rapid development and field evaluation of a portable CRISPR-based assay for Mpox during the 2025 Sierra Leone outbreak
Source: Nat Commun. 2026 Jun 6;17:7241. doi: 10.1038/s41467-026-74034-8 (PMC13396451; doi:10.1038/s41467-026-74034-8)
Supplement: Supplementary file 5 — Reporting Summary [file 41467_2026_74034_MOESM5_ESM.pdf]

nature portfolio

Corresponding author(s): Pardis Sabeti, Nisha Gopal
Last updated by author(s): 05/16/2026

Reporting Summary

Nature Portfolio wishes to improve the reproducibility of the work that we publish. This form provides structure for consistency and transparency in reporting. For further information on Nature Portfolio policies, see our Editorial Policies and the Editorial Policy Checklist.

Statistics

For all statistical analyses, confirm that the following items are present in the figure legend, table legend, main text, or Methods section.

n/a	Confirmed	
☐	☒	The exact sample size (n) for each experimental group/condition, given as a discrete number and unit of measurement
☐	☒	A statement on whether measurements were taken from distinct samples or whether the same sample was measured repeatedly
☐	☒	The statistical test(s) used AND whether they are one- or two-sided<br>*Only common tests should be described solely by name; describe more complex techniques in the Methods section.*
☒	☐	A description of all covariates tested
☒	☐	A description of any assumptions or corrections, such as tests of normality and adjustment for multiple comparisons
☐	☒	A full description of the statistical parameters including central tendency (e.g. means) or other basic estimates (e.g. regression coefficient) AND variation (e.g. standard deviation) or associated estimates of uncertainty (e.g. confidence intervals)
☐	☒	For null hypothesis testing, the test statistic (e.g. F, t, r) with confidence intervals, effect sizes, degrees of freedom and P value noted<br>*Give P values as exact values whenever suitable.*
☒	☐	For Bayesian analysis, information on the choice of priors and Markov chain Monte Carlo settings
☒	☐	For hierarchical and complex designs, identification of the appropriate level for tests and full reporting of outcomes
☐	☒	Estimates of effect sizes (e.g. Cohen's d, Pearson's r), indicating how they were calculated

Our web collection on statistics for biologists contains articles on many of the points above.

Software and code

Policy information about availability of computer code

Data collection	No software was used to collect data in this study. Real-time fluorescence signal measurements were collected either on a commercial DxHub device (DxLab Inc., Somerville, Massachusetts, United States; manufactured under contract by Axxin, Eaglemont, Australia) or on a Biotek Cytation 5 plate reader (Agilent, USA).
Data analysis	ADAPT (Predictive viral design platform, version 1.0, accessible at www.adapt.run); Pathoplexus (Mpox virus sequence database portal, accessed May 2026, version public web interface, www.pathoplexus.org/mpox); GraphPad Prism (version 9.3.0) and Microsoft Excel (version 16.57)

For manuscripts utilizing custom algorithms or software that are central to the research but not yet described in published literature, software must be made available to editors and reviewers. We strongly encourage code deposition in a community repository (e.g. GitHub). See the Nature Portfolio guidelines for submitting code & software for further information.

Data

Policy information about availability of data

All manuscripts must include a data availability statement. This statement should provide the following information, where applicable:

- Accession codes, unique identifiers, or web links for publicly available datasets
- A description of any restrictions on data availability
- For clinical datasets or third party data, please ensure that the statement adheres to our policy

All data supporting the findings of this study are available in the main text or the Supplementary Materials. Source data are provided with this paper. The outbreak-relevant Mpox virus Clade IIb genome sequences used for assay design were sourced from the Pathoplexus database. These public datasets, along with the standard NCBI reference sequence assemblies utilized in this study for alignment and conservation analysis, are permanently available at the following accession codes and hyperlinks:
Outbreak Study Genomes (Pathoplexus):
PP_002XLGK.1 [https://pathoplexus.org/seq/PP_002XLGK.1] (Mpox virus isolate G-29022-1 from Sierra Leone)
PP_002XLHG.1 [https://pathoplexus.org/seq/PP_002XLHG.1] (Mpox virus isolate G-29023-1 from Sierra Leone)
PP_002XKVT.1 [https://pathoplexus.org/seq/PP_002XKVT.1] (Mpox virus isolate G-28995-1 from Sierra Leone)
PP_002XKUV.1 [https://pathoplexus.org/seq/PP_002XKUV.1] (Mpox virus isolate G-28994-1 from Sierra Leone)
PP_002XL0H.1 [https://pathoplexus.org/seq/PP_002XL0H.1] (Mpox virus isolate G-29004-1 from Sierra Leone)
PP_002XKWR.1 [https://pathoplexus.org/seq/PP_002XKWR.1] (Mpox virus isolate G-29000-1 from Sierra Leone)
PP_002XKXP.1 [https://pathoplexus.org/seq/PP_002XKXP.1] (Mpox virus isolate G-29001-1 from Sierra Leone)
PP_002XKSZ.1 [https://pathoplexus.org/seq/PP_002XKSZ.1] (Mpox virus isolate G-28997-1 from Sierra Leone)
PP_002XKTX.1 [https://pathoplexus.org/seq/PP_002XKTX.1] (Mpox virus isolate G-28998-1 from Sierra Leone)
PP_002XLN6.1 [https://pathoplexus.org/seq/PP_002XLN6.1] (Mpox virus isolate G-28999-1 from Sierra Leone)
PP_002XKYM.1 [https://pathoplexus.org/seq/PP_002XKYM.1] (Mpox virus isolate G-29002-1 from Sierra Leone)
PP_002XKZK.1 [https://pathoplexus.org/seq/PP_002XKZK.1] (Mpox virus isolate G-29003-1 from Sierra Leone)
PP_002XL1F.1 [https://pathoplexus.org/seq/PP_002XL1F.1] (Mpox virus isolate G-29005-1 from Sierra Leone)
PP_002XL2D.1 [https://pathoplexus.org/seq/PP_002XL2D.1] (Mpox virus isolate G-29007-1 from Sierra Leone)
PP_002XLBV.1 [https://pathoplexus.org/seq/PP_002XLBV.1] (Mpox virus isolate G-29008-1 from Sierra Leone)
PP_002XL49.1 [https://pathoplexus.org/seq/PP_002XL49.1] (Mpox virus isolate G-29011-1 from Sierra Leone)
PP_002XL57.1 [https://pathoplexus.org/seq/PP_002XL57.1] (Mpox virus isolate G-29012-1 from Sierra Leone)
PP_002XL65.1 [https://pathoplexus.org/seq/PP_002XL65.1] (Mpox virus isolate G-29013-1 from Sierra Leone)
PP_002XL3B.1 [https://pathoplexus.org/seq/PP_002XL3B.1] (Mpox virus isolate G-29010-1 from Sierra Leone)
PP_002XL73.1 [https://pathoplexus.org/seq/PP_002XL73.1] (Mpox virus isolate G-29014-1 from Sierra Leone)
PP_002XLDR.1 [https://pathoplexus.org/seq/PP_002XLDR.1] (Mpox virus isolate G-29015-1 from Sierra Leone)
PP_002XLEP.1 [https://pathoplexus.org/seq/PP_002XLEP.1] (Mpox virus isolate G-29016-1 from Sierra Leone)
PP_002XLFM.1 [https://pathoplexus.org/seq/PP_002XLFM.1] (Mpox virus isolate G-29017-1 from Sierra Leone)
PP_002XL81.1 [https://pathoplexus.org/seq/PP_002XL81.1] (Mpox virus isolate G-29019-1 from Sierra Leone)
PP_002XL9Z.1 [https://pathoplexus.org/seq/PP_002XL9Z.1] (Mpox virus isolate G-29020-1 from Sierra Leone)
PP_002XLAX.1 [https://pathoplexus.org/seq/PP_002XLAX.1] (Mpox virus isolate G-29021-1 from Sierra Leone)
PP_002XLJE.1 [https://pathoplexus.org/seq/PP_002XLJE.1] (Mpox virus isolate G-29025-1 from Sierra Leone)
PP_002XLKC.1 [https://pathoplexus.org/seq/PP_002XLKC.1] (Mpox virus isolate G-29027-1 from Sierra Leone)
PP_002XLLA.1 [https://pathoplexus.org/seq/PP_002XLLA.1] (Mpox virus isolate G-29028-1 from Sierra Leone)
PP_002XLM8.1 [https://pathoplexus.org/seq/PP_002XLM8.1] (Mpox virus isolate G-29029-1 from Sierra Leone)
PP_0031UPN.1 [https://pathoplexus.org/seq/PP_0031UPN.1] (Mpox virus isolate G-29030-1 from Sierra Leone)
PP_0031UQL.1 [https://pathoplexus.org/seq/PP_0031UQL.1] (Mpox virus isolate G-29034-1 from Sierra Leone)
PP_0031URJ.1 [https://pathoplexus.org/seq/PP_0031URJ.1] (Mpox virus isolate G-29036-1 from Sierra Leone)
PP_0031USG.1 [https://pathoplexus.org/seq/PP_0031USG.1] (Mpox virus isolate G-29038-1 from Sierra Leone)
PP_0031UTE.1 [https://pathoplexus.org/seq/PP_0031UTE.1] (Mpox virus isolate G-29040-1 from Sierra Leone)
PP_0031UUC.1 [https://pathoplexus.org/seq/PP_0031UUC.1] (Mpox virus isolate G-29041-1 from Sierra Leone)
PP_0031UVA.1 [https://pathoplexus.org/seq/PP_0031UVA.1] (Mpox virus isolate G-29042-1 from Sierra Leone)
PP_0031UW8.1 [https://pathoplexus.org/seq/PP_0031UW8.1] (Mpox virus isolate G-29043-1 from Sierra Leone)
PP_0031UX6.1 [https://pathoplexus.org/seq/PP_0031UX6.1] (Mpox virus isolate G-29044-1 from Sierra Leone)
PP_0031UY4.1 [https://pathoplexus.org/seq/PP_0031UY4.1] (Mpox virus isolate G-29049-1 from Sierra Leone)
PP_0031UZ2.1 [https://pathoplexus.org/seq/PP_0031UZ2.1] (Mpox virus isolate G-29050-1 from Sierra Leone)
PP_0031V00.1 [https://pathoplexus.org/seq/PP_0031V00.1] (Mpox virus isolate G-29051-1 from Sierra Leone)
PP_0031V1Y.1 [https://pathoplexus.org/seq/PP_0031V1Y.1] (Mpox virus isolate G-29052-1 from Sierra Leone)
PP_0031V2W.1 [https://pathoplexus.org/seq/PP_0031V2W.1] (Mpox virus isolate G-29053-1 from Sierra Leone)
PP_0031V3U.1 [https://pathoplexus.org/seq/PP_0031V3U.1] (Mpox virus isolate G-29054-1 from Sierra Leone)
PP_0031V4S.1 [https://pathoplexus.org/seq/PP_0031V4S.1] (Mpox virus isolate G-29058-1 from Sierra Leone)
PP_0031V50.1 [https://pathoplexus.org/seq/PP_0031V50.1] (Mpox virus isolate G-29059-1 from Sierra Leone)
PP_0031V7L.1 [https://pathoplexus.org/seq/PP_0031V7L.1] (Mpox virus isolate G-29065-1 from Sierra Leone)
PP_0031V8J.1 [https://pathoplexus.org/seq/PP_0031V8J.1] (Mpox virus isolate G-29066-1 from Sierra Leone)
PP_0031V6N.1 [https://pathoplexus.org/seq/PP_0031V6N.1] (Mpox virus isolate G-29061-1 from Sierra Leone)
PP_0031V9G.1 [https://pathoplexus.org/seq/PP_0031V9G.1] (Mpox virus isolate G-29070-1 from Sierra Leone)
PP_0031VAG.1 [https://pathoplexus.org/seq/PP_0031VAE.1] (Mpox virus isolate G-29071-1 from Sierra Leone)
PP_0031VD8.1 [https://pathoplexus.org/seq/PP_0031VD8.1] (Mpox virus isolate G-29110-1 from Sierra Leone)
PP_0031VBC.1 [https://pathoplexus.org/seq/PP_0031VBC.1] (Mpox virus isolate G-29102-1 from Sierra Leone)
PP_0031VE6.1 [https://pathoplexus.org/seq/PP_0031VE6.1] (Mpox virus isolate G-29111-1 from Sierra Leone)
PP_002XLCT.1 [https://pathoplexus.org/seq/PP_002XLCT.1] (Mpox virus isolate G-29009-1 from Sierra Leone)
PP_0031VCA.1 [https://pathoplexus.org/seq/PP_0031VCA.1] (Mpox virus isolate G-29106-1 from Sierra Leone)
PP_0031VF4.1 [https://pathoplexus.org/seq/PP_0031VF4.1] (Mpox virus isolate G-29125-1 from Sierra Leone)
PP_0031VG2.1 [https://pathoplexus.org/seq/PP_0031VG2.1] (Mpox virus isolate G-29126-10) from Sierra Leone)
PP_0031VHZ.1 [https://pathoplexus.org/seq/PP_0031VHZ.1] (Mpox virus isolate G-29127-1 from Sierra Leone)
PP_0031VJX.1 [https://pathoplexus.org/seq/PP_0031VJX.1] (Mpox virus isolate G-29128-1 from Sierra Leone)
PP_0031VKV.1 [https://pathoplexus.org/seq/PP_0031VKV.1] (Mpox virus isolate G-29129-1 from Sierra Leone)
Reference Genomes (NCBI GenBank):
NC_063383 [https://www.ncbi.nlm.nih.gov/nuccore/NC_063383] (Mpox virus Clade IIb reference genome)

NC_006998.1 [https://www.ncbi.nlm.nih.gov/nuccore/NC_006998.1] (Vaccinia virus reference genome)
NC_003663.2 [https://www.ncbi.nlm.nih.gov/nuccore/NC_003663.2] (Cowpox virus reference genome)
AY009089.1 [https://www.ncbi.nlm.nih.gov/nuccore/AY009089.1] (Camelpox virus reference genome)

Research involving human participants, their data, or biological material

Policy information about studies with human participants or human data. See also policy information about sex, gender (identity/presentation), and sexual orientation and race, ethnicity and racism.

Reporting on sex and gender	N/A
Reporting on race, ethnicity, or other socially relevant groupings	N/A
Population characteristics	N/A
Recruitment	Samples were based on archived clinical patient specimens received and stored at Kenema Government Hospital (KGH), which serves as a regional testing and referral center for Mpox diagnostics. Some samples may have been collected at peripheral health facilities and subsequently sent to KGH for confirmatory testing. No recruitment specifications other than expected-positive and expected-negative classification were applied from the investigator perspective. These studies utilized clinical excess specimens; the secondary use of these biological specimens was approved with a waiver of informed consent in accordance with the aforementioned protocols.
Ethics oversight	All research activities complied with relevant ethical regulations and institutional policies. Activities at Kenema Government Hospital (KGH) were conducted under approval from the Sierra Leone Ethics and Scientific Review Committee (protocol 002/05/2024). Activities at the Broad Institute were conducted under approvals from the Harvard Longwood Campus Institutional Review Board (protocols IRB24-0562, IRB24-0563).

Note that full information on the approval of the study protocol must also be provided in the manuscript.

Field-specific reporting

Please select the one below that is the best fit for your research. If you are not sure, read the appropriate sections before making your selection.

- [x] Life sciences
- [ ] Behavioural & social sciences
- [ ] Ecological, evolutionary & environmental sciences

For a reference copy of the document with all sections, see nature.com/documents/nr-reporting-summary-flat.pdf

Life sciences study design

All studies must disclose on these points even when the disclosure is negative.

Sample size	No statistical method was used to predetermine sample size. The clinical sample size (n = 56) was determined by the availability of excess lesion swabs collected at Kenema Government Hospital during the active 2025 Sierra Leone Mpox clade IIb outbreak window. For the pilot evaluation of unextracted samples, a subset of 16 samples (8 qPCR-positive and 8 qPCR-negative) was selected to provide a proof-of-concept for extraction-free workflows. While ideally negative controls would include specimens from patients with other skin-lesions–causing illnesses or infections with related orthopox viruses, such samples were not available during the study period.
Data exclusions	A total of 57 clinical samples were initially provided. One sample was excluded from the final analysis because it returned a borderline qPCR cycle threshold (Ct) of 40, which was deemed of insufficient reliability for a definitive positive reference. No other data were excluded from the analyses.
Replication	All attempts at replication were successful. Reagents during analytical testing were validated across two different platforms (Biotek Cytation 5 and DxHub) to ensure platform-independent reproducibility. Each clinical sample was tested in triplicate to provide statistical rigor, mitigate stochastic effects at low template concentrations, and enable application of replicate-based interpretation rules. For unextracted clinical samples, duplicate reactions were run.
Randomization	The experiments were not randomized, as clinical samples were categorized based on a reference qPCR standard to evaluate diagnostic performance.
Blinding	The investigators were not blinded to allocation during experiments and outcome assessment.

Reporting for specific materials, systems and methods

We require information from authors about some types of materials, experimental systems and methods used in many studies. Here, indicate whether each material, system or method listed is relevant to your study. If you are not sure if a list item applies to your research, read the appropriate section before selecting a response.

Materials & experimental systems

n/a	Involved in the study
☒	☐ Antibodies
☒	☐ Eukaryotic cell lines
☒	☐ Palaeontology and archaeology
☒	☐ Animals and other organisms
☒	☐ Clinical data
☒	☐ Dual use research of concern
☒	☐ Plants

Methods

n/a	Involved in the study
☒	☐ ChIP-seq
☒	☐ Flow cytometry
☒	☐ MRI-based neuroimaging

Plants

Seed stocks	N/A
Novel plant genotypes	N/A
Authentication	N/A
